# Multiomics Analysis of Neuroblastoma Cells Reveals a Diversity of Malignant Transformations

**DOI:** 10.3389/fcell.2021.727353

**Published:** 2021-09-07

**Authors:** Milda Narmontė, Povilas Gibas, Kristina Daniūnaitė, Juozas Gordevičius, Edita Kriukienė

**Affiliations:** ^1^Department of Biological DNA Modification, Institute of Biotechnology, Life Sciences Center, Vilnius University, Vilnius, Lithuania; ^2^Human Genome Research Group, Institute of Biosciences, Life Sciences Center, Vilnius University, Vilnius, Lithuania

**Keywords:** 5-hydroxymethylcytosine, covalent labeling, neuroblastoma, partially methylated domains, cancer biomarkers, hmTOP-seq, TOP-seq, PMD

## Abstract

Neuroblastoma (NB) is a pediatric cancer of the developing sympathetic nervous system that exhibits significant variation in the stage of differentiation and cell composition of tumors. Global loss of DNA methylation and genomic 5-hydroxymethylcytosine (5hmC) is a hallmark of human cancers. Here, we used our recently developed single-base resolution approaches, hmTOP-seq and uTOP-seq, for construction of 5hmC maps and identification of large partially methylated domains (PMDs) in different NB cell subpopulations. The 5hmC profiles revealed distinct signatures characteristic to different cell lineages and stages of malignant transformation of NB cells in a conventional and oxygen-depleted environment, which often occurs in tumors. The analysis of the cell-type-specific PMD distribution highlighted differences in global genome organization among NB cells that were ascribed to the same lineage identity by transcriptomic networks. Collectively, we demonstrated a high informativeness of the integrative epigenomic and transcriptomic research and large-scale genome structure in investigating the mechanisms that regulate cell identities and developmental stages of NB cells. Such multiomics analysis, as compared with mutational studies, open new ways for identification of novel disease-associated features which bring prognostic and therapeutic value in treating this aggressive pediatric disease.

## Introduction

Neuroblastoma is the most common extracranial solid cancer in childhood whose heterogeneous clinical evolution is strongly associated with the cell type composition of NB tumors (Shimada et al., 1999; Ross et al., 2003). A variety of cell types, including cells with neurite-like processes, N-type, fibroblast-like cells, S-type, and cells with intermediate morphology between N- and S-type, I-type, has originally been identified (Ross et al., 2003; Walton et al., 2004). Other recent studies suggested that two types of phenotypically divergent cells, committed adrenergic cells and undifferentiated mesenchymal cells, are dominant in NB tumors (Boeva et al., 2017; van Groningen et al., 2017). Most solid tumors contain a population of mesenchymal stem cells; the hypoxic environment within the tumor attracts mesenchymal stem cells from surrounding stroma which can promote tumor growth and vascularization (Papaccio et al., 2017; Ridge et al., 2017). It has been proposed that in rapidly proliferating NB tumors, inefficient vascular supply causes highly hypoxic regions to appear inducing undifferentiated, more aggressive phenotype of NB leading to adverse clinical outcomes and resistance to therapy (Jögi et al., 2004; Bhaskara et al., 2012).

In human tissues, 5hmC is the most abundant of all known oxidized forms of 5-methylcytosine, whose levels vary in a tissue-specific manner, reaching up to 1.8% of total cytosine in human neurons (Wagner et al., 2015). 5hmC was found at significantly reduced levels in various solid tumors, reinforcing the diagnostic and prognostic significance of 5hmC in cancer research (Haffner et al., 2011; Jin et al., 2011; Kraus et al., 2012; Applebaum et al., 2019). However, our knowledge of the involvement of 5hmC in malignant transformations of phenotypically different NB cell populations in the hypoxic environment is still limited. We, therefore, set out to investigate whether 5hmC is a relevant indicator of aberrant transformations undergoing in various NB cells in the conventional and hypoxic environments. Single-nucleotide resolution bisulfite sequencing using chemical premodification steps has been employed in studies profiling 5hmC distribution in cancer (Chen et al., 2016; Johnson et al., 2016; Raiber et al., 2017; Zhou D. et al., 2018). However, bisulfite treatment causes substantial DNA degradation and requires deep sequencing, which is prohibitively expensive, especially for mapping of cytosine modifications of extremely limited abundance. For comprehensive analysis of genomic 5hmC distribution in NB cells, we used our recently developed bisulfite-free high-resolution hmTOP-seq approach (Gibas et al., 2020).

Cellular transcriptional programs are orchestrated by developmentally regulated mechanisms involving the higher-order organization of chromatin and DNA replication timing. A gene silencing role has been suggested for large partially methylated domains that have been detected in cell lines and tumors (Lister et al., 2009; Berman et al., 2012; Salhab et al., 2018; Brinkman et al., 2019; Decato et al., 2020). In this study, targeting unmethylated CG sites (uCGs) genome-wide by our previously developed uTOP-seq approach (Staševskij et al., 2017) enabled comprehensive capture of PMDs in various NB cell types. We investigated the cell-type-specific PMD distribution, which suggested the apparent PMD involvement in maintaining cell identities, despite the overall variability of PMDs throughout cells and tumors (Brinkman et al., 2019). Our study revealed the diverse roles of 5hmC and general cytosine modification in marking active gene expression which strongly depends on large-scale genome structure, highlighting the interplay of different factors in defining tumorigenicity and developmental stage of NB cells. Moreover, we demonstrated the utility of a large-scale genomic structure for evaluation of the heterogeneity of NB cells.

## Materials and Methods

### Cell Culture

NB cell lines SK-N-BE(2) (ECACC 95011815), BE(2)-C (ECACC 95011817), BE(2)-M17 (ECACC 95011816), LA-N-1 (ECACC 06041201), LA1-5s (ECACC 06041204), and LA1-55n (ECACC 06041203) were maintained in a 1:1 mixture of MEM medium with non-essential amino acids [Thermo Fisher Scientific (TS), Waltham, MA, United States] and Ham’s F12 nutrient mixture (TS) supplemented with penicillin (100 U/ml), streptomycin (100 μg/ml), and 15% fetal bovine serum (FBS) [SK-N-BE(2)] or 15% heat-inactivated FBS [BE(2)-C and BE(2)-M17] or 10% FBS (LA-N-1, LA1-5s, and LA1-55n) at 37°C in atmospheric oxygen concentration (21%) with 5% CO_2_. For hypoxic conditions, cells were incubated at 1% O_2_, 5% CO_2_, and 94% N_2_. Cell cultures were incubated for 24, 48, or 72 h.

### Genomic DNA

DNA from neuroblastoma-cultured cells was purified using standard phenol-chloroform extraction.

### Quantitative Liquid Chromatography Coupled With Tandem Mass Spectrometry Analysis of Genomic DNA

One microgram of gDNA was digested with 0.5 U Nuclease P1 (Sigma, Burlington, MA, United States) for 2 h at 55°C in 42 μl of P1 buffer, then dephosphorylated by adding 1 μl FastAP (TS) phosphatase and incubated overnight at 37°C. Samples were analyzed on an integrated HPLC/ESI-QQQ system (Agilent, Santa Clara, CA, United States) equipped with a Supelco Discovery^®^HS C18 column (7.5 cm × 2.1 mm, 3 μm) by elution with a linear gradient of solvents A (0.0075% formic acid in water) and B (0.0075% formic acid in acetonitrile) at a flow of 0.3 ml/min at 30°C as follows: 0–6 min, 0% B; 6–18 min, 10% B; 18–20 min, 100% B. Mass spectrometer was operating in the positive ion MRM mode, and intensities of nucleoside-specific ion transitions were recorded: d5mC *m*/*z* 242.1→126.1, dhmC *m*/*z* 258.1→142.1, and dG *m*/*z* 268.1→152.1. Ionization capillary voltage was set to 1,800 V, drying gas temperature 300°C and flow rate 10 l/min, collision energy 15 V. Standard d5mC, d5hmC, and dG nucleosides (Trilink Biotech, San Diego, CA, United States) were used for external calibration. Comparison of d5hmC amounts between hypoxic and normoxic cells were performed by Student’s *t* (paired one-tailed analysis) test. *P* < 0.05 was considered as statistically significant. High-performance liquid chromatography coupled with tandem mass spectrometry (HPLC-MS/MS) analysis was performed on four independent experiments.

### Preparation of hmTOP-seq Libraries of NB DNA

Neuroblastoma cells were grown for 72 h at atmospheric or hypoxic conditions, and the extracted gDNA was sonicated on M220 Focused-ultrasonicator (Covaris, Woburn, MA, United States) in 10 mM Tris-HCl (pH 8.5) buffer to yield fragments with a peak size of ∼200 bp. 5hmC glycosylation was carried out in a 100-μl reaction mixture with 800–1,000 ng fragmented gDNA supplemented with 50 μM UDP-6-azide-glucose (Jena Bioscience) and 10 U T4 β-glucosyltransferase (TS) for 2 h at 37°C followed by enzyme inactivation at 65°C for 20 min and column purification [GeneJet PCR Purification kit (TS)]. Azide-tagged DNA was processed as described previously (Gibas et al., 2020). DNA libraries were amplified for 12 cycles, size selected for ∼300 bp fragments (MagJET NGS Cleanup and Size Selection kit, TS), and subjected to Ion Proton (TS) sequencing.

### Preparation of uTOP-seq Libraries of NB DNA

Neuroblastoma cells were grown for 72 h at atmospheric conditions, and the extracted gDNA was sonicated on E220 Evolution Focused-ultrasonicator (Covaris) in 10 mM Tris-HCl (pH 8.5) buffer to yield fragments with a peak size of ∼200 bp. Five hundred nanograms of gDNA were incubated with 0.5 μM eM.SssI in 30 μl 10 mM Tris–HCl (pH 7.4), 50 mM NaCl, 0.5 mM EDTA reaction mixture, supplemented with 200 μM Ado-6-azide cofactor for 1 h at 30°C followed by thermal inactivation at 65°C for 20 min and Proteinase K treatment (0.2 mg/ml) for 30 min at 55°C, and then column purified (DNA Clean & Concentrator-5, Zymo Research, Irvine, CA, United States). After ligation of adaptors to the azide-tagged DNA, alkyne-containing DNA oligonucleotide with biotin was attached, biotinylated DNA was enriched with streptavidin-coupled magnetic beads and subsequently used in a DNA priming reaction as described previously for hmTOP-seq library preparation (Gibas et al., 2020). uTOP-seq DNA libraries were amplified for 11 cycles, size selected for ∼300 bp fragments (MagJET NGS Cleanup and Size Selection kit, TS) and subjected to Ion Proton (TS) sequencing.

### RNA Isolation

Total RNA from NB cells after 72 h incubation under atmospheric or hypoxic conditions was isolated using RNAzol RT reagent according to the manufacturer’s instructions (Molecular Research Center, Cincinnati, OH, United States).

### Quantitative Reverse Transcription PCR

To avoid DNA contamination before RT reaction, RNA samples were treated with dsDNase (TS). Of total RNA, 1.5–2 μg was used for cDNA synthesis in 20 μl reaction mixture supplemented with 1 mM dNTP, 100 pmol Random Hexamer Primer, 20 U RiboLock RNase inhibitor, 200 U RevertAid Reverse Transcriptase (reagents from TS), and incubated for 10 min at 25°C, then for 60 min at 42°C followed by the heat inactivation at 70°C for 10 min. qPCR experiments were performed with a Rotor-Gene Q cycler (Qiagen) using Maxima SYBR Green/ROX qPCR Master Mix (TS). Sequences of primers are provided in Supplementary Table 3. The amplification program was set as 95°C for 10 min and 40 cycles at 95°C for 15 s, 60°C for 30 s, and 72°C for 30 s. Relative mRNA and 3′ncRNA levels were measured by the ΔC_T_ method with normalization to the expression of the endogenous control *B2M* gene. Measurements were independently repeated two times.

### Wound Healing Assay

Cell migration was assessed by a wound healing assay. Cells were grown in 24-well plates in a 1:1 mixture of MEM medium with non-essential amino acids (TS) and Ham’s F12 nutrient mixture supplemented with 1% FBS. Two hundred-microliter plastic pipette tip was used to create a wound in a cell monolayer and images were captured with Primovert microscope (Zeiss, Jena, Germany) at the beginning (0 h) and after 24 and 48 h. The wound area was measured using ImageJ (NIH).

### Preparation of RNA-seq Libraries of NB

mRNA was enriched from the total RNA with Dynabeads mRNA Purification Kit (TS) and ribosomal RNA was depleted using RiboCop rRNA Depletion Kit (Lexogen, Vienna, Austria) according to the manufacturer’s recommendations. cDNA libraries for RNA sequencing were prepared with Ion Total RNA-Seq Kit v2 (TS) in two biological replicates following the manufacturer’s protocol. Final libraries were subjected to Ion Proton (TS) sequencing.

### Analysis of hmTOP-seq Data

Neuroblastoma hmTOP-seq data were processed as described in Gibas et al. (2020) except for read start to CG site distance which was set to 0–3 nt. h-density was calculated using the same algorithm and parameters as for u-density described in Staševskij et al. (2017): first, weighted kernel density estimates of CG coverage were computed using Epanechnikov kernel over 2^21^ points uniformly distributed across each chromosome. Read counts were normalized to sum to 1 and used as weights for the density function. Then, Gaussian kernel smoothing with the same bandwidth was used to interpolate density estimates at CG sites. The same approach, yet with omission of weights was used to estimate the unweighted CG density. Finally, h-density was obtained by dividing weighted CG coverage signal by unweighted CG density at each CG dinucleotide. Kernel bandwidths were 180 bp for coverage weighted density and 80 bp for CG density. This adjustment normalized variation in the library size between different samples and reduced bias of the uneven CG distribution throughout the genome. Enrichment of h-density in genomic elements was calculated by discarding loci with log_2_ h-density below –30 and averaging the remaining signal in non-overlapping regions of 50 bp for each biological replicate. For every genomic element, we used Fisher’s exact test to evaluate the odds ratio of the region overlapping a genomic element and the average h-density of that region above a given *q*th percentile. Hydroxymethylated CH sites were identified as described in Gibas et al. (2020). 5hmCH enrichment in protein-coding genes was calculated using Fisher’s exact test by testing if a cytosine is within a 5hmCH set and overlaps a specific gene. All genes with odds ratio above 1 and *p*-value less than 0.05 were classified as 5hmCH-enriched genes. 5hmCH enrichment within other genomic elements was performed for each CH context separately (i.e., using only cytosines in CA, CT, or CC context) followed by averaging Fisher’s test estimates to obtain a single enrichment value per each element.

### Analysis of RNA-seq Data

RNA-seq reads of BE(2)-C, BE(2)-M17, LA1-55n, and LA1-5s (average number of reads for each cell line was 24 M, *SD* = 4.5 M) were mapped to the reference human genome using STAR aligner (genome build version hg19) (Dobin et al., 2013). STAR aligner was used with default parameters except for “outFilterScoreMinOverLread” and “outFilterMatchNminOverLread” that were set to 0.3. Processed RNA-seq data were normalized in the following way: for each gene type (i.e., protein-coding, lincRNA, pseudogene, 3′ncRNA), all genes that had at least five reads in at least one sample were selected, and read counts from selected genes were globally scaled between feature strata using the upper-quartile method. Then, reads per kilobase per million mapped reads (RPKM) values were computed and log2 transformed for each individual gene (Risso et al., 2011). Epigenome feature influence on gene expression was calculated using a generalized linear model. For each cell line, RNA signal from all expressed genes was selected as a dependent variable while 5hmCH modification status, PMDs, and enhancer elements were used as independent predictors. Gene expression change coefficient was computed according to a baseline, which for 5hmCH genes was genes without 5hmCHs, for genes inside PMDs or on PMD edges—genes outside PMDs, for genes with enhancers—genes that do not overlap enhancer elements. For interaction between 5hmCH genes inside PMDs or present on PMD edges, baseline was genes without 5hmCHs outside PMDs.

### Identification of Differentially Expressed and Hydroxymethylated Genes

Differentially hydroxymethylated regions were identified by first segmenting the genome into 1 kb regions. For each region, we used ANOVA *F*-test to identify regions where the average h-density of a region was less variable within technical replicates than across biological replicates. Regions passing the test with FDR *q* < 0.05 were termed technically reliable and were used for further analysis. For each of the remaining regions, we averaged the signal across technical replicates and fitted a linear model with h-density as the dependent variable and cell type, cell condition, and cytosine ID as the independent variables. ANOVA was used to determine significance of each of the independent variables, and those with FDR *q* < 0.05 were deemed significant. Subsequently, Tukey’s HSD test was applied on selected significant regions to identify the differences between the cell types and conditions.

Differentially hydroxymethylated genes were calculated for each gene type separately using h-density signal averaged per technical replicates. Paired *t*-test was applied for all CG sites within a gene either between the same NB cell type in different conditions (Up- or Down-DHGs) or in the same conditions, but between different NB cell types (cell type-specific DHGs). Genes passing the test with FDR *q* < 0.05 were selected as significant DHGs.

Differentially expressed genes were calculated using the edgeR tool (Robinson et al., 2009). DEGs were calculated either between the same NB cell type for different conditions (Up- or Down-DEGs) or at the same conditions, but different NB cell types (cell type-specific DEGs) with default parameters except “dispersion” that was set to 1e-4. Genes passing the analysis with FDR *q* < 0.05 and log-transformed absolute fold change > 0.5 were selected as significant DEGs.

All enrichment tests: DHRs among various genomic elements, 5hmCGs within gene elements in DEHGs, 3’UTRs of DEHGs with genomic elements, and DEHGs within replication timing groups were performed with Fisher’s exact test. Gene enrichment with SNVs was calculated using 1e6 random sampling permutations. GO enrichment analysis was performed by submitting foreground and background lists of protein-coding genes to GOrilla tool (Eden et al., 2009). Genes mapped to selected significant DEHGs, DHGs, and DEGs were used as the foreground list, whereas all protein-coding genes were used as the background list. Molecular pathway enrichment analysis was performed using the hallmark gene set collection of the Molecular Signatures Database (Subramanian et al., 2005; Liberzon et al., 2015).

### PMD Identification

A sliding window approach was used to identify PMDs in IMR90 and NB cell lines. Each genome was divided into 10 kb non-overlapping windows, and the percentage of uCGs from all CG sites was calculated within each window. A sliding window with a 10-kb increment was used to identify regions having at least 30% uCGs and at least five CGs. All identified PMD windows with a distance below 10 kb were concatenated into a single PMD region. Finally, PMD regions that were larger than 10 kb in size and did not overlap gaps in genome build annotation were selected as a final PMD set. The cell-type-specific PMDs were identified as follows: non-overlapping PMDs between the two subpopulations that contained uCG signal above 0.75 quantile than another cell line were classified as the 2-cell-PMDs. The 4-cell-PMDs were constructed from the 10-kb bins that were uniquely identified in a specific cell line.

For estimation of cellular proportions in the parental NB cell lines, we first prepared the cell mixtures by *in silico* mixing the daughterlines in the following way: CG identification status within a fraction of randomly selected CG sites was swapped between the two daughterlines [BE(2)-M17 and BE(2)-C or LA1-55n and LA1-5s]. This resulted in the changed identification status of some CG sites while in the rest of the CG sites, it remained the same (i.e., when the swapping was performed for identified uCGs or not-identified CGs). This swapping was performed in various fractions with a gradual increase from 1 to 99% with an increment of 1%. These mixtures represented a wide range of daugtherline composition possibilities within a parental cell line. Then, *de novo* PMD regions were identified in these mixtures as specified above. Next, a fraction of uCG sites that were identified in all technical replicates across the unique 2-cell-PMDs was computed (the computation was performed one thousand times using a random subsample of 1% from all original uCG sites). Finally, the computed fractions were averaged to obtain the calibration curves for a fraction of uCG sites in the unique 2-cell-PMDs at a particular cellular mixture. These uCG sites from the 2-cell-PMDs in the mixtures were intersected with uCG sites from the original motherline PMDs (intersection was tested using permutations in a similar way that was mentioned above) to calculate an average daugtherline-specific uCG fraction that can be converted into a fraction of the daughterline within the parental cell line using the calibration curves.

### Annotations

Human genome sequence (build hg19), CGI, and repeat coordinates were downloaded from the UCSC genome browser. CGI shores were defined as the 2-kb regions around a CGI, and CGI shelves were defined as the 2-kb regions around CGI shores. NB-specific typical enhancer sequences were downloaded from the Enhancer Atlas (Gao et al., 2016). Gene dataset was downloaded from GENCODE database (Frankish et al., 2019). Gene upstream (promoter) or downstream regions were set as the 2-kb regions from the given transcription start or end site, respectively. Coordinates of the ADRN and MES S-Enhs and the lists of ADRN and MES mRNA signatures were used from van Groningen et al. (2017). For HIF-1 and HIF-2 regions, we selected only non-overlapping HIF-1 and HIF-2 regions (Schodel et al., 2011). Single nucleotide datasets identified in primary and relapsed NB tumors were taken from Schramm et al. (2015), and SNVs identified in primary NB tumors were taken from Pugh et al. (2013). Replication timing data for SK-N-SH, GM12878, HeLa, IMR90, K562, and MCF-7 cell lines (ENCODE Project; Thurman et al., 2007) were downloaded from the UCSC genome browser. To summarize, replication timing was ascertained by the sequencing-based “Repli-seq” methodology: first, isolation and sequencing of newly replicated DNA from six cell cycle fractions: G1/G1b, S1, S2, S3, S4, and G2 were performed and then, replication patterns were produced as a continuous signal along the genome based on sequencing tag density. We binned this signal into 1-Mb non-overlapping regions and divided into five groups according to signal intensity, where group 1 corresponds to the earliest replication stage and group 5 corresponds to the latest replication stage. For the visualizations, only replication timing data of the NB cell line SK-N-SH were used. uCG signal for PMD calculation in IMR90 was used from Staševskij et al. (2017) and WGBS signal was taken from Lister et al. (2009). Cancer driver genes were downloaded from the catalog of somatic mutations in cancer (Tate et al., 2019). High- and low-risk NB gene sets were taken from Applebaum et al. (2019). They were selected according to the significance (FDR adjusted *p*-values < 0.05) and fold change of the differential 5hmC modification and expression between the two given tumor clusters.

## Results

### Genome-Wide Distribution of 5hmC in NB Cells

We evaluated 5hmC levels in two heterogeneous NB cell lines SK-N-BE(2) and LA-N-1, and their clonal subpopulations, BE(2)-C (I-type) and BE(2)-M17 (N-type), and LA1-55n (N-type) and LA1-5s (S-type), respectively, by an HPLC-MS/MS assay (Supplementary Figures 1A,B). The analysis indicated heavily decreased global levels of 5hmC in each of the samples (0.0025–0.006% of total cytosine) as compared with neural tissues (Globisch et al., 2010; Lister et al., 2013; Wagner et al., 2015). 5hmC amounts increased shortly after the hypoxic exposure (2.5-fold on average), while the 5mC amounts were not affected, consistent with previous reports (Mariani et al., 2014; Thienpont et al., 2016). The hypoxic induction was confirmed by the increased expression of the genes coding for hypoxia-sensitive transcription factors HIF-1α and HIF-2α (Supplementary Figure 2).

**FIGURE 1:**
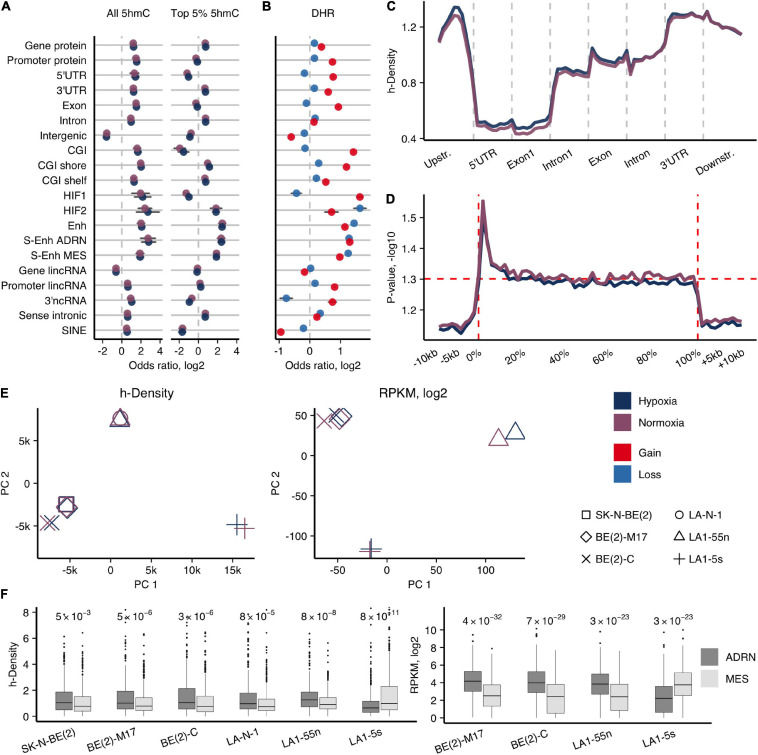
Genome-wide 5hmCG distribution in NB cells. **(A)** Enrichment odds ratios (OR; log2) of the entire and top 5% h-density signals across various genomic elements for the average signal of NB cell lines. Significance obtained using Fisher’s test, only enrichments with *p* < 0.05 reported. **(B)** OR for enrichment of the 1-kb regions with 5hmCG gain and loss (DHRs) across various genomic elements for the average signal of NB cell lines. All *p*-values for reported OR < 1e-7. **(C)** Averaged h-density profile over different gene-associated regions: upstream (2 kb), 5′-untranslated (5′UTR), the first exon and intron, other exons and introns, 3′-untranslated (3′UTR), and downstream (2 kb) regions. **(D)** ANOVA along the protein-coding genes and the upstream, downstream regions (–10 kb, +10 kb) for all NB cell lines shows the significant h-density difference along the whole gene body, with the highest difference right after the transcription start site (0% corresponds to TSS, and 100% to TTS). **(E)** Principal component analysis of h-density (left) and RNA-seq data (right). **(F)** Distribution of h-density (left) and expression levels (right) across the genes of the ADRN and MES signatures (van Groningen et al., 2017). *p*-values obtained from two-sided paired *t*-test.

We established genome-wide hypoxic and normoxic 5hmCG maps of all NB cell lines (for sequencing statistics see Supplementary Table 1). On average, 1.67 M (5.9%, *SD* = 1.8%) genomic CGs were identified in two technical replicates of the hmTOP-seq libraries for each of the NB cell lines (≥5x coverage, Supplementary Figure 3A) which correlated well (Pearson mean *r* = 0.88, *SD* = 0.05) (Supplementary Figure 3D). The single-base readout of hmTOP-seq also allowed identification of 5hmC in a non-CG context - up to 30 thousand 5hmCH sites where H corresponds to A, T or C (average coverage 3.4x; correlation between hmTOP-seq library replicates Pearson mean *r* = 0.85, *SD* = 0.03). The numbers of 5hmCG/5hmCH sites and their coverage rose under hypoxia, as expected (Supplementary Figures 3A, 4). To eliminate CG density effect on hmTOP-seq CG read count across various regions, we calculated normalized density (h-density), as described for uTOP-seq (Staševskij et al., 2017), and used it further for determination of differently hydroxymethylated genes (DHGs) or genomic elements. h-density increased the average correlation between technical replicates up to 0.95 (Supplementary Figures 3B,C). 5hmCGs and h-density signals were similarly enriched across genomic elements, attesting the strongest signal (top 5%) in NB-defined typical enhancers (data of SK-N-SH NB cell line, Gao et al., 2016) and super enhancers (data of van Groningen et al., 2017) [Fisher’s test log2 odds ratio (OR) = 1.21 – 3.6, *p* < 2.2e-16], introns, 3′UTRs, CGI shores and shelves, and HIF-2 hypoxia response elements (Schodel et al., 2011), whereas intergenic regions were depleted of 5hmCGs (Figure 1A). Among non-protein coding genes, we observed an enrichment for sense intronic non-coding RNAs, gene 3′-end overlapping non-coding RNAs (3′ncRNA), and promoters and genes of long intergenic non-coding RNAs. We noted a sharp contrast in h-density enrichment between processed and unprocessed pseudogenes (Pei et al., 2012): the highest h-density levels were attested in transcribed processed pseudogenes (Supplementary Figure 5), confirming the relationship of 5hmC with active expression (Wu et al., 2011; Wen and Tang, 2014).

To evaluate which regions undergo 5hmC dynamics in hypoxia, we calculated the h-density changes in 1-kb regions genome-wide using the average signal of all NB cells (5hmC gain and loss regions, DHRs) (Figure 1B). 5hmC gain regions were more likely to be found in CG islands and shores (Fisher’s test log2 OR range = 1.2–1.4, *p*-values < 2.2e-16), exons, and all types of enhancers (Fisher’s test log2 OR range = 1–1.4, *p*-values < 2.2e-16). Assessment of the h-density distribution across the composite gene revealed an increasing signal toward the 3’-end of the gene with the strongest signal in the 3’UTR region and a relatively high h-density in the upstream region (Figure 1C). To search for the most variable h-density signal across the gene-associated elements, we conducted ANOVA analysis along the protein-coding genes, and, surprisingly, found a significant difference along the whole gene body with the highest variability in the areas flanking TSS (Figure 1D). Therefore, we further used the combined h-density signal of the whole gene body for calculations of the protein-coding genes that gain 5hmC in hypoxia. The common hypoxic 5hmC gain set of all NB cell lines included the genes of various epigenetic or chromatin remodeling enzymes (DNMT3A, DNMT3L, KDM4B, KDM6B, HDAC4, etc.) and proteins regulating development (NOTCH and WNT pathway proteins, the members of SOX, PAX, KLF families, including the pluripotency maintaining factors, SOX2 and KLF4) (Supplementary Table 2), suggesting that hypoxia affects key epigenetic players and molecular processes that control cellular differentiation.

In agreement with the knowledge that NB tumors are mostly composed of the N-type cells (Ross et al., 1995; Walton et al., 2004), the parental SK-N-BE(2) and LA-N-1 cell lines clustered with the respective N-type subpopulations in principal component analysis of h-density signal, while S-type LA1-5s and I-type BE(2)-C cells diverged from the other cell lines in their groups, indicating their different 5hmC signatures (Figure 1E). To compare 5hmC and expression signatures of different NB cell types, we performed RNA-seq of the four subpopulations of SK-N-BE(2) and LA-N-1. PCA of RNA-seq signals again demonstrated a distinction among the subpopulations of SK-N-BE(2) and LA-N-1 (Figure 1E). Recent analysis identified two main cell populations of NB tumors as adrenergic (ADRN) and undifferentiated mesenchymal/neural crest-like cell phenotypes (MES/NCC-like) (Boeva et al., 2017; van Groningen et al., 2017). Evaluation of expression and h-density levels of the ADRN (358 genes) and MES (479 genes) genes (van Groningen et al., 2017) classified all used NB cell lines as ADRN, except for LA1-5s that showed associations with the MES/NCC-like signature (Figure 1F).

### Hypoxia-Dependent 5hmC and Expression Signatures of Different NB Cell Types

Hypoxic changes were first tested using 30 known hypoxia response genes (Schofield and Ratcliffe, 2004). The majority of them, including the well-known HIF-1α/HIF-2α-regulated genes, such as, *BNIP3*, *ENO1*, *GPI*, *HK2*, *PGK1*, and *VEGFA*, showed a coordinated increase in h-density (∼68% of genes, *SD* = 8%) and expression (∼72% of genes, *SD* = 6%) in all NB cells, except for LA1-5s (Supplementary Figure 6). We confirmed the correlation between 5hmC and enhanced gene activity, using the RNA-seq data of SK-N-BE(2) (Mariani et al., 2014) (mean 69%, *SD* = 7%; single-sided paired *t*-test *p* = 8.3e-5), and by the quantitation of *VEGFA* expression in the SK-N-BE(2) group cells (Supplementary Figure 2).

**FIGURE 2:**
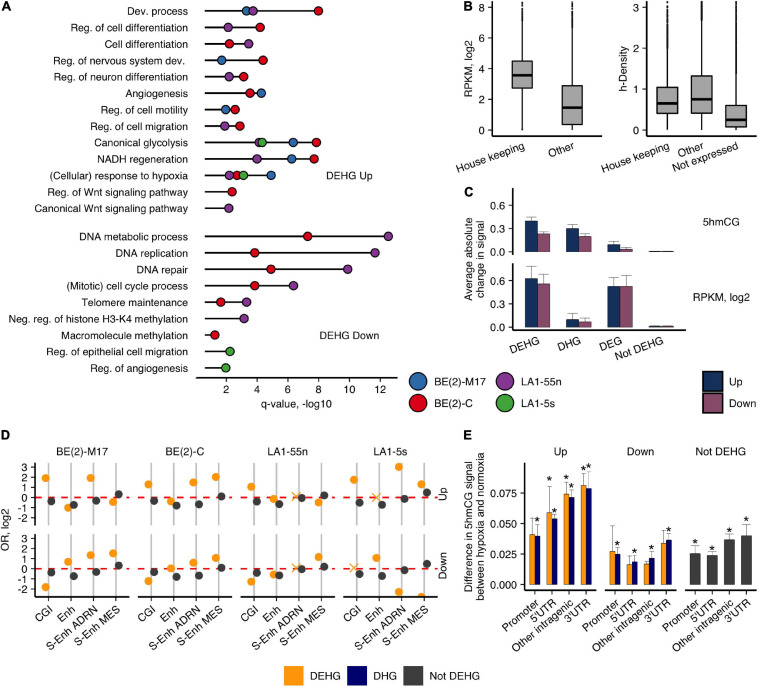
5hmCGs distributed differently in expressed genes of hypoxic and normoxic NB cells. **(A)** GO functional annotation analysis of the hypoxic Up- and Down-DEHG with 5hmCG gain and loss, respectively. Reg., regulation; Dev., development; Proc., process; Diff., differentiation. *q*-value represents enrichment significance after Benjamini and Hochberg correction. **(B)** h-density and RNA levels for the housekeeping genes in relation to other expressed and unexpressed genes combined for all NB cell lines (*p*-values between housekeeping and other genes for both comparisons were < 2.2e-16, Wilcoxon signed-rank test). **(C)** Absolute average change of the 5hmCG (upper panel) and RNA (bottom panel) signals between the up and down subgroups of DEHGs, DHGs, DEGs, and not DEHGs (genes without expression or 5hmCG changes). **(D)** OR for enrichment of the 3′UTRs of the protein-coding genes for CGIs and NB-defined Enhs (data of SK-N-SH NB cell line, Gao et al., 2016) and S-Enhs (van Groningen et al., 2017). Non-significant estimates (*p* ≥ 0.05) are marked with “X.” **(E)** Difference in the average 5hmCG fractions between hypoxia and normoxia in promoter, 5′UTR, intragenic and 3′UTR CGIs of Up-DEHGs, Down-DEHGs, and not DEHGs for all NB cell types. Asterisks above the bars indicate significant 5hmCG differences (*p*-value < 0.05, *t*-test) between hypoxia and normoxia.

To evaluate the hypoxic gene signatures, we divided the protein-coding genes into three groups: (1) genes that gained or lost 5hmC, Up-DHGs, and Down-DHGs, respectively; (2) genes with induced or repressed expression, Up-DEGs, and Down-DEGs; (3) genes that showed concomitant changes in both 5hmCGs and expression, Up-DEHGs, and Down-DEHGs, respectively (Supplementary Figure 7). We performed GO functional annotation analysis of all three groups (Figure 2A and Supplementary Figure 8). Up-DHGs and Up-DEHGs showed high association with development-related and cell identity-specific processes, whereas Up-DEGs were mainly linked to metabolic processes, signaling, and other housekeeping functions. Although housekeeping genes (3,718 genes, Eisenberg and Levanon, 2013) are more extensively expressed than other genes in NB cells (all *p*-values < 2.2e-16) (Figure 2B), they contain lower h-density levels, suggesting that other mechanisms regulate their expression. Up-DEHGs/DHGs of all NB subpopulations except of LA1-5s showed links to regulation of cell differentiation, nervous system development, and cell migration, whereas Down-DEHGs/DHGs were related to DNA metabolism, DNA replication, and repair. In contrast, DNA metabolism, replication, and repair-associated genes were not downregulated by hypoxia in the S-type LA1-5s. As the highest hypoxia-induced change in both 5hmCG and RNA levels was observed in DEHGs in comparison with genes without significant 5hmCG or expression changes (not DEHG) (Figure 2C), we focused our further analysis mainly on this group.

For protein-coding genes, the highest 5hmCG levels were detected at their 3′UTRs (Figure 1A). Therefore, we were interested to explore whether this is influenced by their overlap with regulatory elements. The 3′UTRs of Up-DEHGs most significantly overlapped with CGIs, NB-specific Enhs and ADRN/MES S-Enhs (Figure 2D). All NB cell types showed enrichment for ADRN S-Enhs, while BE(2)-C and LA1-5s also demonstrated the enrichment for MES S-Enhs, pointing to some similarity between these cells. Considering that CGIs showed the strongest 5hmCG gain in hypoxia (Figure 1B), we compared the 5hmCG fractions across 5′UTR, promoter and intragenic CGIs of DEHGs. The 5hmCG fraction increased most in 3′UTR CGIs of Up-DEHGs as compared with 5′UTR, promoter and intragenic CGIs, while in Down-DEHGs and not DEHGs the 5hmCG changes at all types of CGIs remained the same (Figure 2E). GO analysis of Up-DEHGs (and Up-DHGs) with increased 5hmCG levels across 3′UTR CGIs indicated a strong association with cell differentiation (e-8 to e-12, all cell types); regulation of transcription by RNA polymerase II (e-5 to e-15, all cell types), neuron differentiation (e-7 to e-9, all cell types); Wnt signaling pathway (2.8e-2 and 7.7e-3 for LA1-55n and BE(2)-C cells, respectively), further indicating that the genes participating in cellular developmental programs in NB undergo hypoxic 5hmCG changes at regulatory regions.

Of all types of tested non-coding RNAs, 3′UTR overlapping 3′ncRNAs demonstrated hypoxic 5hmCG increase in our NB cell lines (Figure 1B). We observed expression and increased 5hmCG levels in seven 3′ncRNAs (of 21 annotated 3′ncRNAs): *RP11-571M6.8*, *AC012442.5*, *AC064852.4*, *CTC-510F12.4*, *AC010729.1*, *AC092620.2*, and *RP5-1126H10.2*. We further tested the three most expressed 3′ncRNAs, *AC010729.1*, *RP5-1126H10.2*, and *CTC-510F12.4*, as they indicated potential cell-type specificity, and their overlapping protein-coding genes by quantitative reverse transcription PCR (RT-qPCR) (Supplementary Figure 9A). The expression of *AC010729.1*, *RP5-1126H10.2*, and their host genes *SOX11* and *UBR4*, respectively, was elevated in both N-type cells BE(2)-M17 and LA1-55n, while *CTC-510F12.4* or its host gene *RAB3D* was more expressed in all three ADRN cells. To explore the cell identity-defined regulation of these 3′ncRNAs and their host genes, we searched for the presence of the lineage-specific S-Enhs in the surrounding areas. The localization of an ADRN S-Enh was detected in the 274-kb distance to the *SOX11* gene, the host of *AC010729.1*, pointing to the involvement of these genes in neuronal lineage-dependent processes, which are more advanced in N cells. All tested genes were less intensively expressed in LA1-5s while only *CTC-510F12.4* and its host *RAB3D* showed increased expression in BE(2)-C, as compared with LA1-5s, again suggesting a similarity between these cells. We detected the MES S-Enhs within 100 kb–1 Mb distances to *UBR4* and *RAB3D*, indicating that these genes might be specifically regulated by the MES-specific S-Enh network.

### 5hmC Defines Cell Type-Specific Features and Malignant Transformations

**FIGURE 3:**
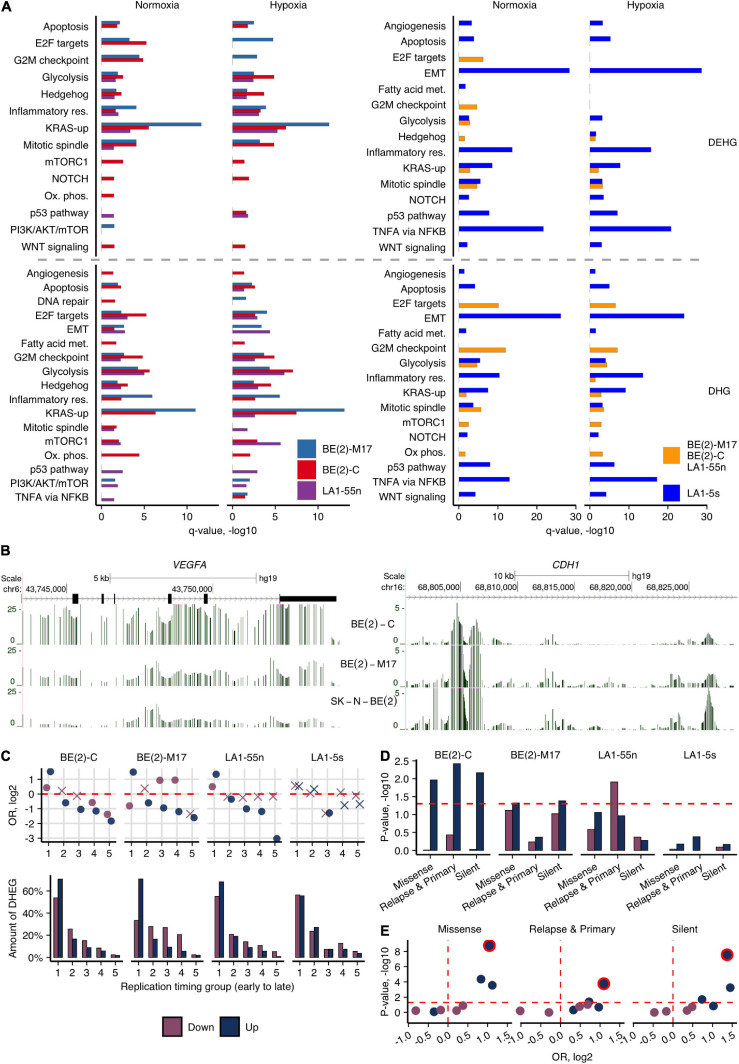
Cell type-specific 5hmCG differences of NB cells. **(A)** Molecular pathway enrichment analysis of DEHGs and DHGs identified by comparing each of the ADRN cells [BE(2)-C, BE(2)-M17, and LA1-55n] against the MES/NCC-like LA1-5s cells or between LA1-5s and all three ADRN cells combined, in normoxia and hypoxia. Significance of pathway enrichment is represented by *q*-value. Met., metabolism; Res., response. **(B)** Browser representation of h-density profiles across the loci of *CDH1* and *VEGFA* genes in hypoxic cells of the SK-N-BE(2) group shows the decreased and increased signal in BE(2)-C, respectively. **(C)** OR from Fisher’s exact test and fractions of Up- and Down-DEHGs across genomic regions, which were assigned numbers from 1 to 5 based on replication timing (1 and 5 represent early and late replication timing groups, respectively. The replication timing data of SK-N-SH was used for the visualizations). Non-significant estimates (*p* ≥ 0.05) are marked with “X.” **(D)** Significance of the overlap between Up- and Down-DEHGs and the three sets of SNVs identified in NB tumors (Pugh et al., 2013; Schramm et al., 2015). Enrichment *p*-values were calculated from 1e6 permutations. **(E)** Statistical significance (*p*-value) and OR values for Fisher’s test enrichment of NB-specific SNVs across Up- and Down-DEHGs of four NB subpopulations that overlap early replicating regions (1st replication timing group). The most significantly enriched BE(2)-C cells were outlined.

We next explored how 5hmC reveals epigenetic differences of various hypoxic and normoxic NB cells and variations in differentiation stages, which might contribute to their different tumorigenicity. The analysis of DHGs determined the distinctive molecular pathway signatures of BE(2)-C cell population (I-type) when compared with BE(2)-M17 (N-type) of the same tumor origin (Supplementary Figure 10). Furthermore, we identified DEHGs and DHGs by comparing the subpopulations of potentially different identities, i.e., the ADRN subpopulations BE(2)-C, BE(2)-M17, and LA1-55n against the MES/NCC-like LA1-5s cells, in normoxia and hypoxia, and analyzed their molecular pathway associations (Figure 3A). The analysis recapitulated the previous trend for BE(2)-C to be in a less differentiated state (Supplementary Figure 10) and pointed to its stemness characteristics. mTORC1, Notch, and Wnt pathways were uniquely enriched in BE(2)-C in both conditions (Figure 3A, DEHGs). BE(2)-C also demonstrated active proliferation (E2F targets, G2M checkpoint in normoxia, and mitotic spindle in hypoxia) and metabolism, the latter including both glycolysis and oxidative phosphorylation as energy sources (data of DHGs/DEHGs). Genes involved in the PI3K/AKT/mTOR axis signaling and, more specifically, mTOR complex 1 (mTORC1) signaling, primarily functioning as an energy/nutrient sensor and protein synthesis regulator, were upregulated in BE(2)-C (compared with BE(2)-M17, Supplementary Figure 10). Furthermore, BE(2)-C were 5hmC depleted across the genes participating in neural stem cell differentiation (e.g., *ASCL1*, *BDNF*, *MYT1L*, *POU3F2*), embryonic differentiation and the epithelial-mesenchymal transition (EMT), such as *CDH1* coding for the tumor suppressor *E*-cadherin. It is known that downregulation of *E*-cadherin by the epidermal growth factor receptor EGFR and the main regulator of EMT, SNAI1, is a prominent hallmark of cancer invasion (Bremm et al., 2008; Wang et al., 2013). In addition, the gene of a vascular endothelial growth factor VEGFA, which can promote cancer progression by its involvement in angiogenesis and cell migration, is highly expressed and hydroxymethylated in BE(2)-C (Figure 3B). Elevated 5hmC and expression levels of *SNAI1*, *EGFR*, and *VEGFA* in hypoxia and repression of *CDH1* (Figure 3B) most likely indicate the increased aggressiveness of BE(2)-C cells. Testing the cell migratory abilities indeed revealed the elevated migration potential of BE(2)-C in relation to BE(2)-M17 (Supplementary Figure 9B). Moreover, we observed the enrichment of the BE(2)-C-specific genes for Hedgehog signaling (Figure 3A and Supplementary Figure 10), which plays key roles in proliferation of adult stem cells, facilitating the appearance of cancer stem cells (Papaccio et al., 2017). In contrast, analysis of both DHGs and DEHGs of the N-type BE(2)-M17 revealed enrichment in KRAS signaling, indicating the involvement of different pathways in NB progression.

The tissue of origin for NB is neural crest (Johnsen et al., 2019). In line with the suggested MES/NCC-like identity of LA1-5s, DEHGs/DHGs of LA1-5s were associated with cell migration, motility, and cell adhesion (Figure 3A and Supplementary Figure 11)—all characteristic to EMT and neural crest cell migration, whereas DEHGs of all ADRN subpopulations overlapped with development, neurogenesis, and neuron differentiation (GO analysis; Supplementary Figure 11). Additionally, beside its high migration abilities (Supplementary Figure 9B), LA1-5s showed links to TNFα signaling *via* NF-κB, NOTCH, KRAS, and WNT/β-catenin signaling, angiogenesis, etc., which, importantly, were enriched independently of the cultivation conditions. It is known that TNFα, NF-κB, and WNT pathways crosstalk to regulate EMT (Du and Geller, 2010; Wu and Zhou, 2010) which together with WNT and NOTCH signaling are crucial for neural crest development and NCC migration (Mead and Yutzey, 2012; Ji et al., 2019).

To further account for potential reasons that could induce variance in different tumorigenicity of NB cell types, we explored the link between 5hmC-defined gene expression signatures and common single-nucleotide variations (SNV) in relation to replication timing. Replication timing has a prominent role for accumulation of genetic changes in cancer cells: mutations in late-replicating regions, which include less intensively expressed cell-specific or silent genes, are more likely to be tolerated by a cell than mutations of growth- and development-related genes that usually tend to replicate early (Maric and Prioleau, 2010; Woo and Li, 2012). The NB cells show differences in proliferation intensity, with BE(2)-C cells having the shortest population doubling time (ATCC and our data) and demonstrating significant enrichment of DEHG/DHGs in replication-related processes (Figure 3A and Supplementary Figure 10). As the hypoxic environment could contribute to increased tumor progression (Muz et al., 2015) and, as shown above, affects development- and replication-related genes in NB cells, we first explored the distribution of hypoxic Up- and Down-DEHGs across five replication timing groups (using the replication timing data of six different cell lines including the NB cell line SK-N-SH, see section “Materials and Methods”). The results indicated the association of the hypoxic Up-DEHGs of ADRN cells, BE(2)-C, BE(2)-M17, and LA1-55n, with early replication (Figure 3C). Furthermore, the cell-type-specific DEHGs of BE(2)-C tended to localize in the most early replicating regions (Supplementary Figure 12). We then calculated the enrichment of the hypoxia-affected DEHGs for SNVs often found across protein-coding genes in NB tumors (Pugh et al., 2013; Schramm et al., 2015; 95% of SNVs found outside of CG dinucleotides). Importantly, the most significant overlap with SNVs was observed for Up-DEHGs of BE(2)-C that tend to replicate early (Figures 3D,E and Supplementary Figure 13). We suppose that hypoxia induces early replicating cancer driver genes of BE(2)-C (Fisher’s OR of the enrichment of Up-DEHGs for the common cancer driver genes is 1.6, *p*-value < 0.05) which due to their higher replication rate are more mutation prone and thus, may facilitate cancer development in the hypoxic environment. None of the LA1-5s-specific DEHG sets significantly overlapped with any type of SNVs nor showed enrichment for early replication areas, indicating the low potential for malfunctioning, despite the *MYCN*-amplified status of LA1-5s genome.

### Landscape of Large-Scale Genomic Structures Predicts NB Cell Identity Differences

To get deeper insights into global epigenomic transformations of various NB cell types, we next explored the distribution of large-scale genomic structures, called PMDs. Previously, we visualized PMDs in NB cell lines which overlapped well PMDs detected by whole-genome bisulfite sequencing (WGBS) in IMR90 cell line (Lister et al., 2009; Staševskij et al., 2017). To refine the uTOP-seq parameters for detection of PMDs, we calculated uCG fractions and compared them with CG methylation data of IMR90 across the 10-kb bins genome-wide. Both data corresponded well (Supplementary Figure 14) and suggested the 30% uCG fraction as a threshold; using this threshold, we observed nearly all (98%) PMDs larger than >100 kb and a significant fraction of smaller, <100 kb, PMDs (90%) reported in IMR90. Importantly, the calculation of uCG fractions involves counting uCGs irrespective of their sequencing coverage, and thus, can yield consistent PMDs even from low coverage data (2–4 × coverage in uTOP-seq). This novel strategy for identification of PMDs by uTOP-seq detected more PMDs in IMR90 than WGBS (8,088 vs. 8,682 PMDs that span 1,260 and 1,418 Mb, respectively) and identified two clusters in each PMD size group, which both fulfill the criteria of PMDs but differ in global methylation levels (Supplementary Figure 15).

**FIGURE 4:**
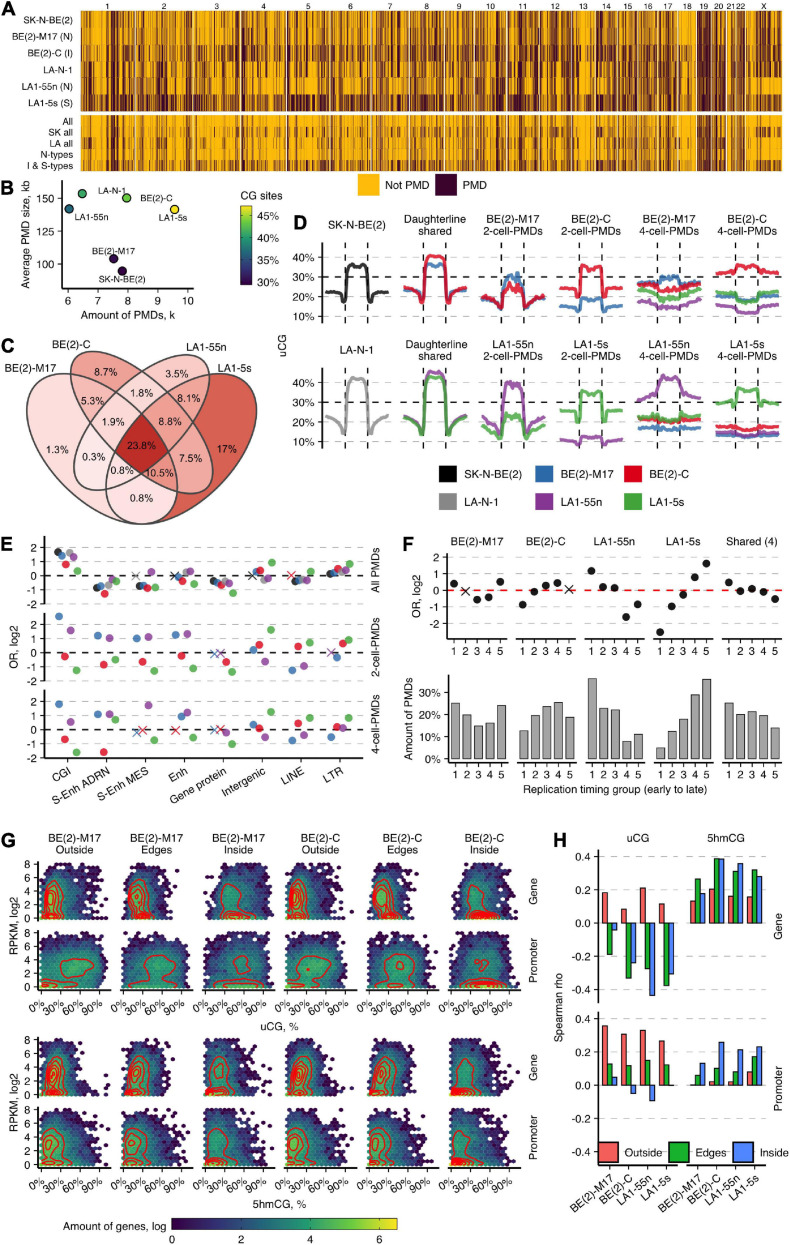
Large-scale genome organization of NB cell types predicts cell identity. **(A)** Genome-wide maps of PMD distribution in normoxic NB cell lines. Below are shown the maps of the overlapping PMD regions for the indicated cell lines. SK all and LA all represent the SK-N-BE(2) and LA-N-1 groups, respectively. **(B)** Distribution of PMD amounts (in thousands, k) and their average sizes in NB cell lines. **(C)** Venn diagram for the overlap of PMDs in 10-kb tiles for the subpopulations of the SK-N-BE(2) and LA-N-1 groups. Percentage indicates the amount of tile overlap between given subpopulations. **(D)** uCG profiles across PMDs and 50-kb upstream and downstream regions of the parental cell lines SK-N-BE(2) and LA-N-1; PMDs shared between the subpopulations of the SK-N-BE(2) and LA-N-1 groups; the unique 2-cell-PMDs; and the unique 4-cell PMDs identified among the four subpopulations. The line at 30% indicates a uCG threshold used for PMD identification. PMDs were divided into 20 equally sized non-overlapping windows and average uCG fractions were computed for each given cell line. **(E)** OR from Fisher’s exact test for enrichment of all PMDs and the unique 2-cell-PMDs and 4-cell-PMDs across various genomic features. Non-significant estimates (*p* ≥ 0.05) are marked with “X.” **(F)** Enrichment OR and amounts of the unique 2-cell-PMDs and the shared for all four subpopulation PMD bins (23.8%, **C**) in five replication timing regions. **(G)** Heatmaps and two-dimensional density estimates for uCG/5hmCG fraction and expression data distributions for genes localized outside of PMDs (Outside), within PMDs (Inside), and crossing PMD boundaries (Edges) in BE(2)-M17 and BE(2)-C. **(H)** Spearman’s correlation between uCG or 5hmCG fractions and RNA data as a function of gene location within or outside of PMDs or overlapping PMD boundaries. Promoters are defined as the 2-kb regions upstream TSS.

Next, by measuring uCG fractions in the consecutive tiles of 10 kb, we generated PMD maps of NB cell lines. All six NB cell lines displayed the widespread loss of DNA methylation, with more extensive PMDs in the LA-N-1 cell group and BE(2)-C (Figures 4A,B). PMDs span up to 990 Mb (*SD* = 240, 33%) on average of the genome, covering from 30 to 47% of genomic CG sites. BE(2)-C and LA1-5s differ most from the other NB cell subpopulations: larger PMDs (≥100 kb) constituted ∼20% of all PMDs in these cell types, while in both N-type subpopulations, this amount was only 6%. The chromosome-wide view of PMD distribution showed the considerable diversity among all six NB cell lines and highlighted higher similarity between BE(2)-C and LA1-5s cells. The overlap of PMDs in 10-kb tiles among the NB subpopulations revealed the common ∼24% fraction and again showed stronger overlap between BE(2)-C and LA1-5s (8.02%) (Figure 4C). Given the overall low numbers of common PMD tiles, we next compared the shared PMDs between the subpopulations of the LA-N-1 and SK-N-BE(2) groups. We noticed a large PMD expansion in BE(2)-C and LA1-5s, which ranged from 40 kb in LA1-5s to 90 kb in BE(2)-C on average across different PMD size groups. The observed similarities between LA1-5s and BE(2)-C probably points to the MES/NCC-like features or a less differentiated state of BE(2)-C.

### PMD Distribution Characterizes the NB Cell Identities and Controls the Cell-Type-Specific DEHGs

We next sought to explore the informativeness of PMDs to define NB cell identities. As expression and 5hmC signature analysis suggested identity differences among the NB subpopulations, we searched for the unique PMDs between the subpopulations of the LA-N-1 and SK-N-BE(2) groups (2-cell-PMDs) and among all four subpopulations (4-cell-PMDs). As expected, BE(2)-C and LA1-5s contained considerably higher numbers of 2-cell-PMDs and 4-cell-PMDs as compared with the corresponding N-type cells [2-cell-PMDs: 2,815, BE(2)-C; 443, BE(2)-M17 and 685, LA1-55n; 4,576, LA1-5s; 4-cell-PMDs: 3,568, BE(2)-C; 898, BE(2)-M17 and 1,710, LA1-55n; 4,673, LA1-5s]. The unique PMDs differed in their features from the shared PMDs: they were much smaller in size (average PMD size for the unique BE(2)-C and LA1-5s PMDs was 74 kb, while for their N-type counterparts—45 kb; the average size of the shared PMD was 160 kb) and less pronounced, considering from the profiles (Figure 4D). Despite the global PMD size differences, the distribution of the unique PMDs segregated both N-type cells from BE(2)-C and LA1-5s: N-type PMDs showed enrichment for CGIs and enhancer regions, whereas PMDs of BE(2)-C and LA1-5s were depleted for almost all tested genomic elements, except of intergenic areas, lincRNAs, LTR, and LINE families (Figure 4E). The similarity between the unique PMDs of both N-type cells or LA1-5s and BE(2)-C was also confirmed by their distinctive distribution according to replication timing: PMDs of both N-type cell lines tended to distribute in early replicating areas, whereas those of LA1-5s and BE(2)-C showed increased enrichment for late replicating regions (Figure 4F). The common for the four NB subpopulations PMD tiles (23.8%; see Figure 4C) were enriched in early replicating regions, which might indicate that NB-associated cells employ PMD-directed silencing of some genes localized in early replicating and usually most actively transcribed regions (Supplementary Figure 16). Notably, replication timing can influence transcription frequencies within the unique PMDs, thereby controlling the known transcription silencing role of PMDs; we found that genes present in early replicating regions of the unique PMDs can be equally highly expressed as genes outside of PMDs, and thus, escape PMD silencing effect which becomes more prominent only for late replicating genes (Supplementary Figure 17).

To assess DNA modification of different expression groups within and outside of PMDs, we integrated uCG and 5hmCG fraction and gene expression data (Figure 4G). We categorized all genes into those localized outside (Outside group) or within PMDs (Inside group) or overlapping PMD boundaries (Edges group; 40% of the all PMD-associated genes). For the Outside group, a cluster of more intense expression showed mean 17% 5hmCGs and 12% uCGs across gene bodies and strongly unmethylated promoters (mean 45% uCGs, 7% 5hmCGs), while the second cluster of mostly silenced genes contained uCG- and 5mCG-depleted promoters (mean 5% 5hmCGs and 3% uCGs) and more unmethylated gene bodies (mean 15% uCGs). For the Inside group, genes distributed mostly in a single cluster of lowly expressed or silent genes depleted of gene body modification (mean 42% uCGs; 7% 5hmCGs) which are hypervariable across their promoter uCG fraction. However, a fraction of PMD-associated genes with increased promoter and gene body hydroxymethylation (mean 5hmCGs 17%) manage to escape PMD-directed silencing. The Edges groups demonstrated the intermediate distribution, with most of the genes showing expression comparable with the outside genes. Of note, the Edges groups of the unique 2-cell-PMDs of both N-type cells displayed more intense expression as compared with BE(2)-C and LA1-5s cells (Supplementary Figure 18). Correlation between CG site modification and expression showed the distinct roles of uCGs and 5hmCGs in regulation of genes within/outside PMDs: promoter and gene body 5hmCG levels contribute most to gene activation within PMDs, while high promoter uCG fraction marks increased expression outside of PMDs (Figure 4H). This points out that, in contrast to the suggested repression function of promoter 5hmC (Wu et al., 2011), it plays a dual role that is controlled by large-scale genomic structure.

**FIGURE 5:**
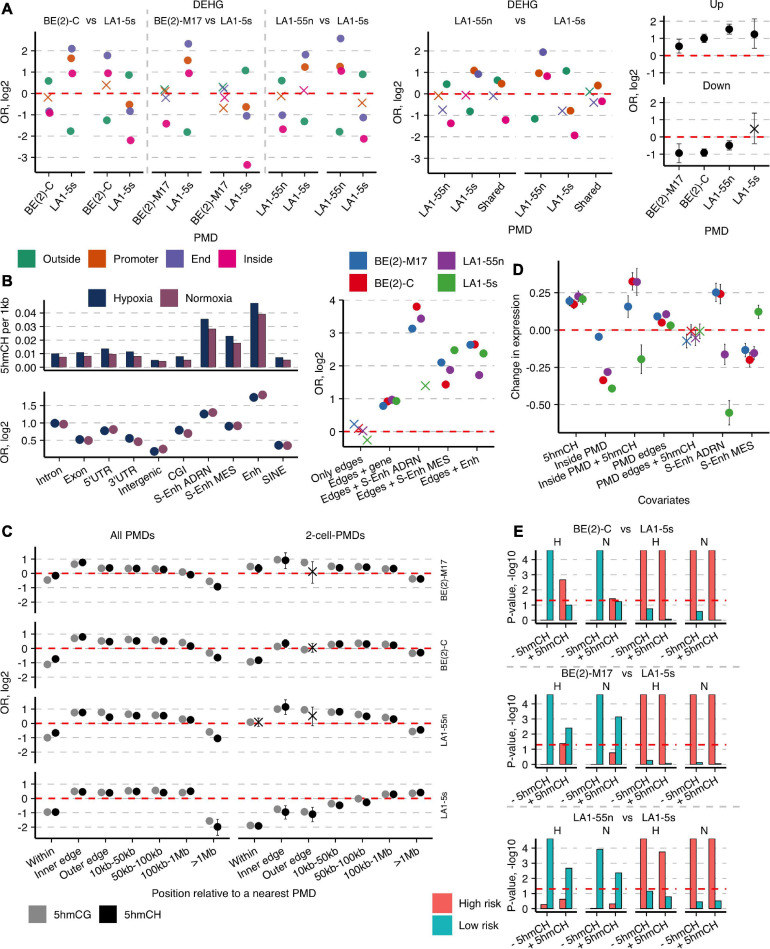
Hydroxymethylated CH and CG dinucleotides mark important enhancers and cell type-specific genes and concentrate around PMDs. **(A)** OR from Fisher’s exact test for enrichment of the cell type-specific DEHGs across End, Promoter, and Inside groups of the unique 4-cell-PMDs (left) or 2-cell-PMDs (middle panel) and OR for enrichment of the hypoxia-affected Up- and Down-DEHGs (right) of each NB cell line across their own PMDs. **(B)** Left, numbers of 5hmCHs (per 1 kb) and OR from Fisher’s exact test for enrichment of 5hmCH sites across various genomic features in NB cell lines. Non-significant estimates (*p* ≥ 0.05) are marked with “X.” Right, OR for enrichment of 5hmCH sites at PMD boundaries or PMD boundaries that overlap specific genomic elements—typical Enhs, ADRN and MES S-Enhs, and protein-coding genes. **(C)** Distribution of enrichment OR values of 5hmCHs and 5hmCGs at all PMDs or the 2-cell-PMDs and their boundaries. Inner and outer edges are represented by the 3-kb regions flanking both sides of PMD boundaries. **(D)** Weight coefficients representing the effect of 5hmCHs, PMDs, typical Enhs and ADRN and MES S-Enhs, and their combinations on gene expression. Coefficient estimates, standard errors, and significance were computed using a generalized linear model (*p*-values ≥ 0.05 are marked with “X”). **(E)** Significance represented as permutation *p*-values (100 k permutations) of the overlap between the cell-type-specific DEHG groups split according to the 5hmCH enrichment status (- and +5hmCH) and the published DEHGs of NB tumors stratified according to patient survival and high-risk (HR) and low-risk (LR) tumors (Applebaum et al., 2019). “H” and “N” define hypoxia and normoxia, respectively. Significance threshold of *p*-values < 0.05 is presented with a horizontal line.

To better envision the regulatory functions of the cell-type-specific PMDs, we next explored the distribution of the DEHG groups as a function of their localization within/outside PMDs. For deeper analysis, the Edges group was split according to the direction of genes—promoters and up to 20% of gene bodies are covered by PMDs in the Promoter group, whereas in the End group, PMDs overlap 20–40% of gene 3′-end parts. Strikingly, the cell-type-specific DEHGs identified between the subpopulations of potentially different cell identities, i.e. BE(2)-C/BE(2)-M17 and LA1-5s/LA1-55n, tend to position across the regions which overlap the boundaries (mostly End groups) of the unique PMDs of the other cell line compared, strongly suggesting a regulatory role of PMDs in maintaining NB cell specificity (Figure 5A, left and middle). Indeed, GO analysis of PMD-associated genes of BE(2)-M17 revealed links with cell adhesion (e-4), regulation of RNA metabolic process (e-3), spindle assembly (e-2), whereas LA1-5s PMDs associated with cell adhesion (e-10), regulation of trans-synaptic signaling (e-8) and nervous system development (e-7) (all data of the 4-cell-PMDs). The different trend was observed for the hypoxic DEHGs: up-DEHGs of each subpopulation were significantly enriched across their own PMDs (Figure 5A, right), suggesting that hypoxia might activate genes controlled by PMDs (if assumed that PMD distribution does not substantially differ between normoxic and hypoxic NB cells).

The cell-type specificity of PMDs inspired us to explore their utility for evaluation of cellular proportions in the parental heterogeneous NB cell lines SK-N-BE(2) or LA-N-1. By measuring PMD overlap between the daughterlines and the corresponding motherlines, we found that SK-N-BE(2) or LA-N-1 are mainly composed of the two daughter subpopulations: only 7 and 11% of all PMDs were uniquely present in SK-N-BE(2) and LA-N-1, respectively. To imitate the subpopulations mixed at different ratios, we *in silico* created the mixtures using CG identification status that represented a wide range of daugtherline composition possibilities within a parental cell line, and then, identified *de novo* PMDs and uCG fractions in each cell mixture. The uCG fractions of the unique 2-cell-PMD regions were averaged to obtain the calibration curves for a uCG fraction at a particular cellular mixture (Supplementary Figure 19). Next, by comparing the uCG fractions of the 2-cell-PMDs detected in the motherlines and the cellular mixtures, we estimated equal proportions of LA1-55n and LA1-5s within LA-N-1, and approximately 1:3 ratio of BE(2)-C and BE(2)-M17 cells, respectively, in SK-N-BE(2).

### 5hmCH-Enriched Enhancers and Genes Delineate PMDs

As 5hmC outside CG context rarely occurs in mammalian genomes, its distribution and functional role were underexplored, especially due to the lack of a simple 5hmCH detection technique. hmTOP-seq enabled identification of a significant number of 5hmCH sites in NB cells (Supplementary Figure 4). 5hmCHs were most significantly enriched at enhancers and genes, mainly introns (Figure 5B), and tended to distribute in the coding strand (Fisher’s test OR from 1.1 to 1.27, *p*-values < 0.022). 5hmCHs at introns were mostly distributed in CA dinucleotides (the ratio of CA:CT:CC is 4:3:3). The observed localization of 5hmCH sites in NB cells corresponds to the known accumulation of methylated non-CG sites in gene body regions and inactive enhancers in mammalian cells, which most likely are introduced by DNMT3A and DNMT3B (He and Ecker, 2015) and oxidized by TET proteins. Of note, we observed the increased density of 5hmC in both CH and CG dinucleotides around PMD boundaries: while 5hmC signal is depleted inside PMDs, its enrichment increases toward PMD boundaries with a peak within a 3-kb region at the inner boundary side, then gradually diminishes and disappears at the >1-Mb distance from PMDs (Figure 5C). Importantly, 5hmCH and 5hmCG distribution demonstrated the strong cell-type specificity; while the unique PMDs of both N-type cells showed 5hmC enrichment, the LA1-5s PMDs were 5hmC-poor, and the BE(2)-C PMDs presented the intermediate enrichment score (Figure 5C). Moreover, PMD boundaries displayed enrichment in 5hmCH-containing genes, Enhs and ADRN/MES S-Enhs, the latter showing distribution in a cell identity-specific manner (Figure 5B, right). GO functional analysis further revealed the cell-type-specific functions of the 5hmCH-containing genes: processes related to neuron projection guidance, regulation of cell projection organization, and anatomical structure development (*q*-values at e-7) were enriched for both N cells, whereas the common 5hmCH genes of BE(2)-C and LA1-5s cells showed links to positive regulation of cellular processes (e-5), extracellular structure organization (e-4), and regulation of signal transduction (e-4). Importantly, we found the preferential enrichment of the cell type-specific DEHGs in 5hmCHs (average Fisher’s test OR for DEHGs 4.7, for not DEHGs 0.3; *p*-values < 10-e7). As genic 5hmCH and 5hmCG amounts are correlated (Supplementary Figure 20), 5hmC in both CG and CH contexts reflects epigenetic regulation processes undergoing at cell identity genes of NB cells.

As only a fraction of protein-coding genes contains 5hmCHs in NB cells (on average 850 genes vs. 18,200 genes with 5hmCGs), we next constructed a generalized linear model to assess the impact of 5hmCHs on gene expression. The model revealed a positive influence of 5hmCHs on expression and showed the strong upregulation, similar to enhancers, for genes localized within PMDs in the cells with ADRN characteristics, BE(2)-M17, BE(2)-C, and LA1-55n (Figure 5D). The analysis also validated the strong and cell identity-specific impact of ADRN/MES S-Enhs and, importantly, evidenced the distinct regulation of gene expression around PMDs, as expression levels at PMD boundaries exceeded those of outside PMDs. Taken together, 5hmC in combination with large-scale genomic organization are important regulators of neuronal lineage-specific genes whose functions are dynamically controlled across developmental stages.

We next sought to explore the link of 5hmCHs with patient outcomes. We used the published DEHG groups, acquired by hMe-Seal approach that enriches the aggregate 5hmCG and 5hmCH signal, of the two NB tumor cohorts stratified according to patient survival, high-risk (HR) and low-risk (LR) tumors (Applebaum et al., 2019), and calculated their overlap with the normoxic and hypoxic cell-type-specific DEHG groups, split according to the 5hmCH enrichment status (Figure 5E). The influence of 5hmCHs was most prominent for the DEHGs of hypoxic BE(2)-C: 5hmCHs enhanced their association with HR genes, whereas the 5hmCH-depleted DEHGs of BE(2)-C as well as those of both N-type cell lines most significantly overlapped with LR group in both conditions. In hypoxic BE(2)-C cells, 5hmCH level was elevated in *DOT1L* gene, which is known to promote NB proliferation (Wong et al., 2017). Additionally, the 5hmCH-enriched genes of BE(2)-C, such as collagens (*COL5A1*, *COL13A1*, *COL23A1*), cytoskeletal proteins (*MYH9*, *PLEC*) (Piskareva et al., 2015), TGFβ, and SHH signaling pathway members (*TGFB1*, *GLI2*) (Shao et al., 2017) could contribute to EMT induction and tumor invasiveness. Surprisingly, the DEHGs of the non-tumorigenic MES/NCC-like LA1-5s cells significantly related to the HR dataset in all comparisons.

## Discussion

As whole-genome sequencing and data analysis still remains hardly accessible to large-scale population and clinical studies, cost-effective sensitive techniques are in high demand for unlocking the diagnostic and prognostic potential of the DNA modification marks in cancer treatment. We demonstrated the extreme informativeness of our previously developed hmTOP-seq and uTOP-seq approaches, both based on covalent derivatization of epigenetic sites and *in situ* primed amplification of adjacent genomic regions (TOP-seq strategy), for single-base resolution analysis of genomic DNA modifications. Both approaches offer unprecedented cost-efficiency; hmTOP-seq resulted in very informative 5hmCG and 5hmCH datasets as demonstrated by mapping the whole human hydroxymethylome at an average of 5 × coverage using only 15 M of processed reads. Moreover, our approach to measure uCG fractions by uTOP-seq independently of sequencing coverage allowed construction of a PMD landscape of NB cells from shallow sequencing data (30 M of processed reads, 2–3 × coverage). Most importantly, this study provided the integrative analysis of 5hmC and expression data in relation to large-scale epigenomic structures of various NB cell types, which highlighted regulatory functions of 5hmC and PMDs in essential epigenetic processes of this developmental disease and cell identity maintenance.

The comprehensive 5hmC analysis of NB cells in hypoxia evidenced the dynamics of genes regulating cellular development and cell lineage identities, beside common metabolic responses to oxygen deficiency. Despite the predicted ADRN identity of the N-type BE(2)-M17 and I-type BE(2)-C cells of the same tumor origin, the cell type-specific 5hmC, 3′ncRNA, and protein-coding gene expression signatures featured the subtle differences between these cells and provided evidence for supporting the shared MES/NCC-like characteristics of the S-type LA1-5s and BE(2)-C. It is known that hypoxia induces the response of molecular mechanisms facilitating migration of neural crest cells (Scully et al., 2016; Niklasson et al., 2020). Accordingly, the population of LA1-5s cells preserves its MES/NCC-like characteristics and migratory ability regardless of oxygen supply in cultivation conditions. The analysis suggested the high tumorigenic potential of BE(2)-C: of BE(2)-M17 and BE(2)-C cells, BE(2)-C were characterized as the most actively replicating NB cells in normal and oxygen-deficient conditions. Additionally, BE(2)-C displayed 5hmC enrichment in the genes involved in the mitotic spindle assembly (Figure 3A and Supplementary Figure 10), which is directly associated with aneuploidy frequently observed in tumor cells. As DNA repair processes are impaired in hypoxia (Figure 2A and Supplementary Figure 8), all these alterations in BE(2)-C could favor the observed epigenetic malfunctioning and mutational potential of developmental genes, contributing to the tumorigenicity of the BE(2)-C subpopulation and aggressiveness of NB.

The observed elevated 5hmCG levels in non-coding regulatory 3′ncRNAs boosted their expression analysis which reported the cell-type specificity of 3′ncRNAs and their host genes, and suggested similarity between the two N-type cells or BE(2)-C and LA1-5s. As information on the roles of 3′ncRNAs in cancer is scarce (*RP11-571M6.8* was detected in neural tumors, Deng et al., 2018), the host genes of the analyzed 3′ncRNAs participate in cancer progression-related processes. The *UBR4* gene, a host of *RP5-1126H10.2*, is upregulated in a metastatic breast tumor cell line, and its inhibition suppresses the invasiveness of gastric cancer (Montel et al., 2005; Sakai et al., 2011). RAB3D, a host of *CTC-510F12.4*, induces EMT in breast cancer cells (Yang et al., 2015). Moreover, RAB3D was detected in tumor-derived exosomes, including those of SK-N-BE(2) cells, suggesting its associations with tumor invasion (Fonseka et al., 2019). Therefore, the roles of these 3′ncRNAs and their host genes in NB development require further detailed investigation.

Analysis of PMDs along with 5hmC distribution provided a more complete picture of NB cell identities. In this study, we focused on the cell-type-specific PMDs as opposed to common PMDs which are large, gene-poor, late-replicating, and often shared among multiple cancers and tissue types (Lister et al., 2009; Berman et al., 2012; Salhab et al., 2018; Zhou W. et al., 2018; Brinkman et al., 2019; Decato et al., 2020). We hypothesize that cell-type-specific PMDs are not just a consequence of late-replicating state of a genome which has not yet achieved full methylation during proliferation, as suggested (Zhou W. et al., 2018), but perform functions indispensable to establishment of cell identity. Even though PMDs are proposed to be a nearly universal feature of actively proliferating cells, the abundance of PMDs in slowly proliferating MES/NCC-like LA1-5s points to the cell identity as a major moderator of PMD distribution. We showed that PMD distribution, even better than 5hmC, serves as cell-type classifiers, highlighting the common features of large-scale genomic structure of the MES/NCC-like LA1-5s and ADRN BE(2)-C cells, and demonstrating similarities between both ADRN N-type cells, despite the overall PMD variability in these cells. Notably, the distribution of the cell-type-specific PMDs of both N-type cell lines in early replicating areas, in contrast to the late replicating unique PMDs of BE(2)-C and LA1-5s, further classified the ADRN BE(2)-M17 and BE(2)-C of the same tumor origin to different developmental stages or identity groups.

Despite the known gene silencing role of PMDs, expression of genes within the cell-type-specific PMDs is regulated in a replication timing-dependent manner and can approach levels of those outside PMDs while more prominent gene silencing is observed in late replicating areas. We suggest that hypoxia might activate developmentally regulated genes in PMDs (Figure 5A), as the hypoxia-affected and cell-type-specific DEHGs tend to be enriched across early replicating areas (Figure 3C), which include genes essential for cell development processes (Dellino et al., 2013). Generally, the multiomics analysis demonstrated the distinct role of promoter 5hmC in expression upregulation of PMD-associated genes: increased promoter and gene body 5hmCG levels mark genes that manage to escape PMD silencing, whereas high promoter uCG-fraction is a main indicator of expression outside of PMDs. Moreover, the 5hmC-enriched cell-type-specific genes were found to be distributed within genomic loci prone to the cell-type-specific PMD regulation, emphasizing the active 5hmC dynamics at lineage-identity genes.

Finally, this study focused on the distribution and impact of non-CG hydroxymethylation on gene expression in NB. We found that 5hmCHs along with 5hmCGs concentrate at regions surrounding PMDs and are enriched at the cell-type-specific PMDs of both N-type cells. Moreover, 5hmCHs predominantly occur within the cell-type-specific DEHGs and enhancers which are enriched at PMD boundaries. We suppose that genomic loci of active 5hmC-enriched cell identity genes and enhancers might stop spreading of passive demethylation and accumulation of PMDs. Our analysis showed that 5hmCHs enhance gene expression in NB cells, and strikingly, may upregulate the PMD-associated genes with the strength equal to enhancers (Figure 5D). Importantly, we found that the 5hmCH-containing cell-specific gene set of hypoxic BE(2)-C and, preferentially, the gene sets of LA1-5s cells, which both display MES/NCC-like characteristics, showed associations with poor survival of NB patients. Although the abundance of the fibroblast-like S cells in NB tumors suggests more favorable disease prognosis (Mora et al., 2001), we assume that migratory abilities of the non-tumorigenic S-type LA1-5s might enhance invasive behavior of NB cells, similarly as recently shown for S-type cells that could promote viability of N cells (Li et al., 2021).

To our knowledge, this is the first report revealing PMD topology and high-resolution 5hmC patterns of various NB cell types, which are often used as cell models. NB, similarly to other pediatric cancers, has a remarkably low genetic complexity (Schramm et al., 2015). This study provided a link between the intrinsic nature of different NB cell lineages and their propensity for malignant transformations, highlighting the importance of a multilevel analysis in understanding the prognostic significance of epigenomic data for harnessing the extensive cellular heterogeneity of NB tumors. We also demonstrated the potential to utilize the cell-type-specific PMD sets for evaluation of the cellular compositions of heterogeneous NB cell lines and tumors. It is known that different NB cell types can interconvert into each other (Ross et al., 2003; van Groningen et al., 2017), resulting in the appearance of transitional cell states whose PMD topology might differ from their precursors. Therefore, construction of a comprehensive set of cell identity-specific PMDs of all NB cell types or states would be of high importance for evaluation of cellular heterogeneity of NB tumors.

## Data Availability Statement

The datasets presented in this study can be found in online repositories. The names of the repository/repositories and accession number(s) can be found below: Gene Expression Omnibus GSE130612.

## Author Contributions

EK coordinated the experimental design of the hmTOP-seq and uTOP-seq, analytical procedures, and genomic analyses. MN established the hmTOP-seq protocol for NB cells, performed the RT-qPCR and cell migration assays, prepared the HPLC-MS/MS samples and analyzed the data, and prepared the hmTOP-seq, uTOP-seq, and RNA-seq libraries. MN and KD performed the molecular pathway and GO analysis of the cell-type-specific data. JG consulted on calculations of DHR, PCA, and expression models. PG established all data analysis pipelines, carried out the bioinformatic and statistical analyses, and performed visualizations. EK, MN, and PG wrote the manuscript with input or comments from all authors. All the authors contributed to the article and approved the submitted version.

## Conflict of Interest

The authors declare that the research was conducted in the absence of any commercial or financial relationships that could be construed as a potential conflict of interest.

## Publisher’s Note

All claims expressed in this article are solely those of the authors and do not necessarily represent those of their affiliated organizations, or those of the publisher, the editors and the reviewers. Any product that may be evaluated in this article, or claim that may be made by its manufacturer, is not guaranteed or endorsed by the publisher.

## Abbreviations

3′ ncRNA: non-coding RNA which overlaps 3’UTR of a protein-coding gene
5hmC: 5-hydroxymethylcytosine
5hmCG: hydroxymethylated CG site
5hmCH: hydroxymethylated non-CG site where H corresponds to A, T, or C
5mC: 5-methylcytosine
ADRN: adrenergic neuroblastoma cells
CGI: CG island
DEG: differentially expressed gene
DEHG: differentially expressed and hydroxymethylated gene
DHG: differentially hydroxymethylated gene
DHR: differentially hydroxymethylated region
Down-DEG: genes with repressed expression in hypoxia
Down-DEHG: genes with decreased both expression and hydroxymethylation in hypoxia
Down-DHG: genes with reduced hydroxymethylation in hypoxia
EMT: epithelial-mesenchymal transition
Enh: typical enhancer
GO: gene ontology
h-density: normalized 5hmC density
HIF: hypoxia-inducible factor
hmTOP-seq: 5hmC-specific tethered oligonucleotide-primed sequencing
HR: high risk
k: thousand
lincRNA: long intergenic non-coding RNA
LINE: long interspersed nuclear element
LR: low risk
LTR: long terminal repeat
M: million
MES: mesenchymal neuroblastoma cells
NB: neuroblastoma
NCC: neural crest cell
OR: odds ratio
PCA: principal component analysis
PMD: partially methylated domain
RT-qPCR: quantitative reverse transcription PCR
SD: standard deviation
S-Enh: super-enhancer
S-Enh ADRN: super-enhancer of the ADRN cell lineage identity
S-Enh MES: super-enhancer of the MES cell lineage identity
Sense intronic: sense intronic non-coding RNA
SNV: single-nucleotide variant
TSS: transcription start site
TTS: transcription termination site
uCG: unmodified CG site
Up-DEG: genes with induced expression in hypoxia
Up-DEHG: genes with elevated both expression and hydroxymethylation in hypoxia
Up-DHG: genes with increased hydroxymethylation in hypoxia
uTOP-seq: uCG-specific tethered oligonucleotide-primed sequencing
UTR: untranslated region
WGBS: whole-genome bisulfite sequencing.
