# Supplementary material for: Multiomics Analysis of Neuroblastoma Cells Reveals a Diversity of Malignant Transformations
Source: Front Cell Dev Biol. 2021 Sep 7;9:727353. doi: 10.3389/fcell.2021.727353 (PMC8452964; doi:10.3389/fcell.2021.727353)
Supplement: Supplementary file 1 [file Data_Sheet_1.pdf]

Supplementary Figures

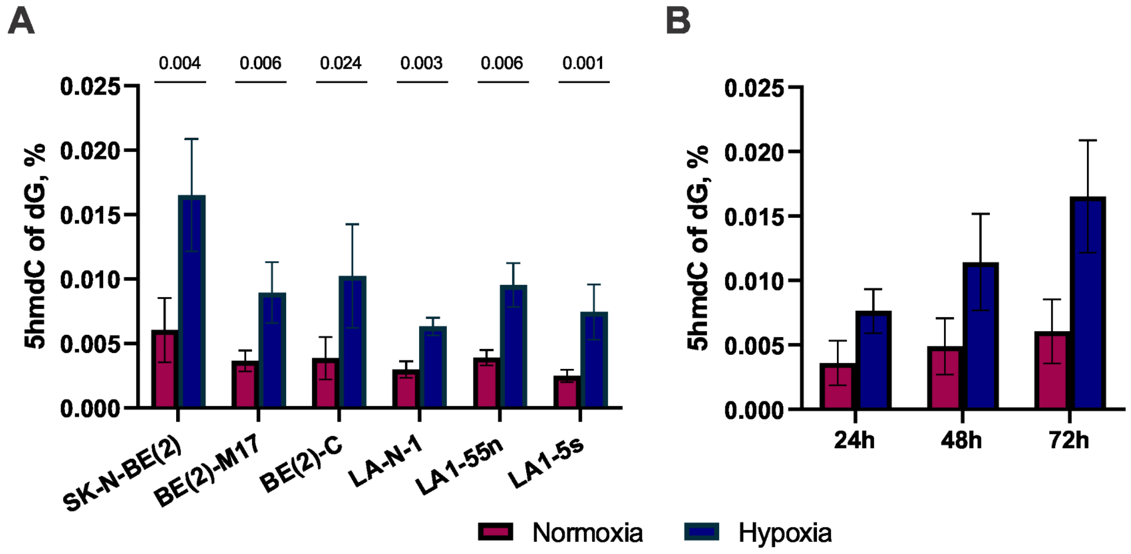

Supplementary Figure S1. HPLC-MS/MS quantitation of 5hmC in NB cell lines. **(A)** 5hmC amounts in the parental NB cell lines SK-N-BE(2) and LA-N-1, and their clonal subpopulations BE(2)-C (I-type), BE(2)-M17 (N-type), and LA1-55n (N-type), LA1-5s (S-type), respectively, cultivated in atmospheric or hypoxic conditions (72 h of hypoxia) (n = 4). Data represents mean ± sd. Values above the bar plots indicate p values obtained using single-sided paired t-test. **(B)** Timeline of 5hmC increase in SK-N-BE(2) exposed to 24 h, 48 h and 72 h of hypoxia (n = 4). Data represents mean ± sd.

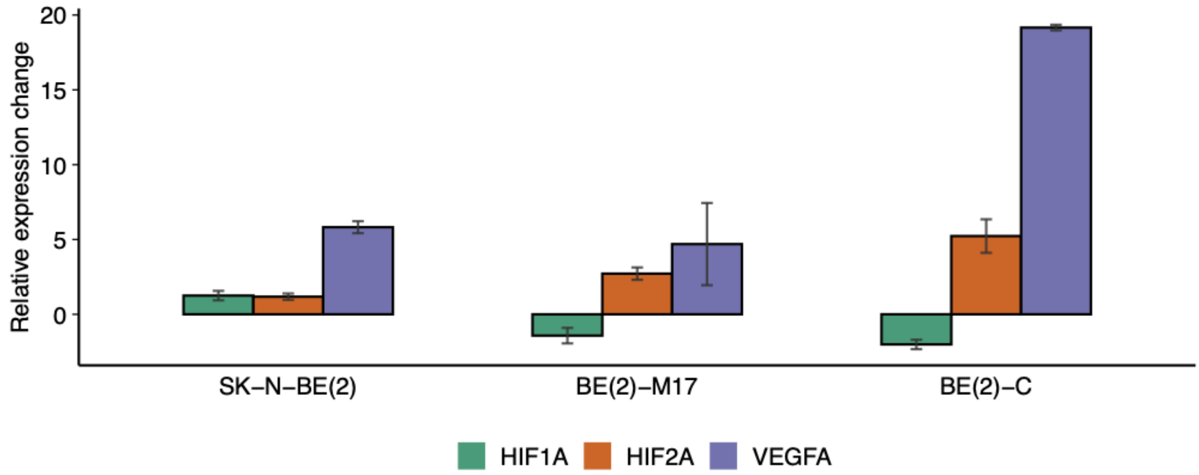

Supplementary Figure S2. Quantification of *HIF1A, HIF2A* and *VEGFA* mRNA by quantitative reverse transcription PCR (RT-qPCR) after 72 h of hypoxia in the SK-N-BE(2) group-related cells. Data represents means ± sd of two experiments. The *HIF2A* gene coding for the hypoxia-sensitive transcription factor HIF-2α after 72 h of hypoxia showed an increased expression in all tested NB cell lines, while *HIF1A* coding for HIF-1α showed a weak or undetectable induction. This is in agreement with previous observations that HIF-1α

primarily mediates a fast response and HIF-2α mediates late responses during hypoxic adaptation (Holmquist-Mengelbier et al. 2006; Jögi et al. 2004).

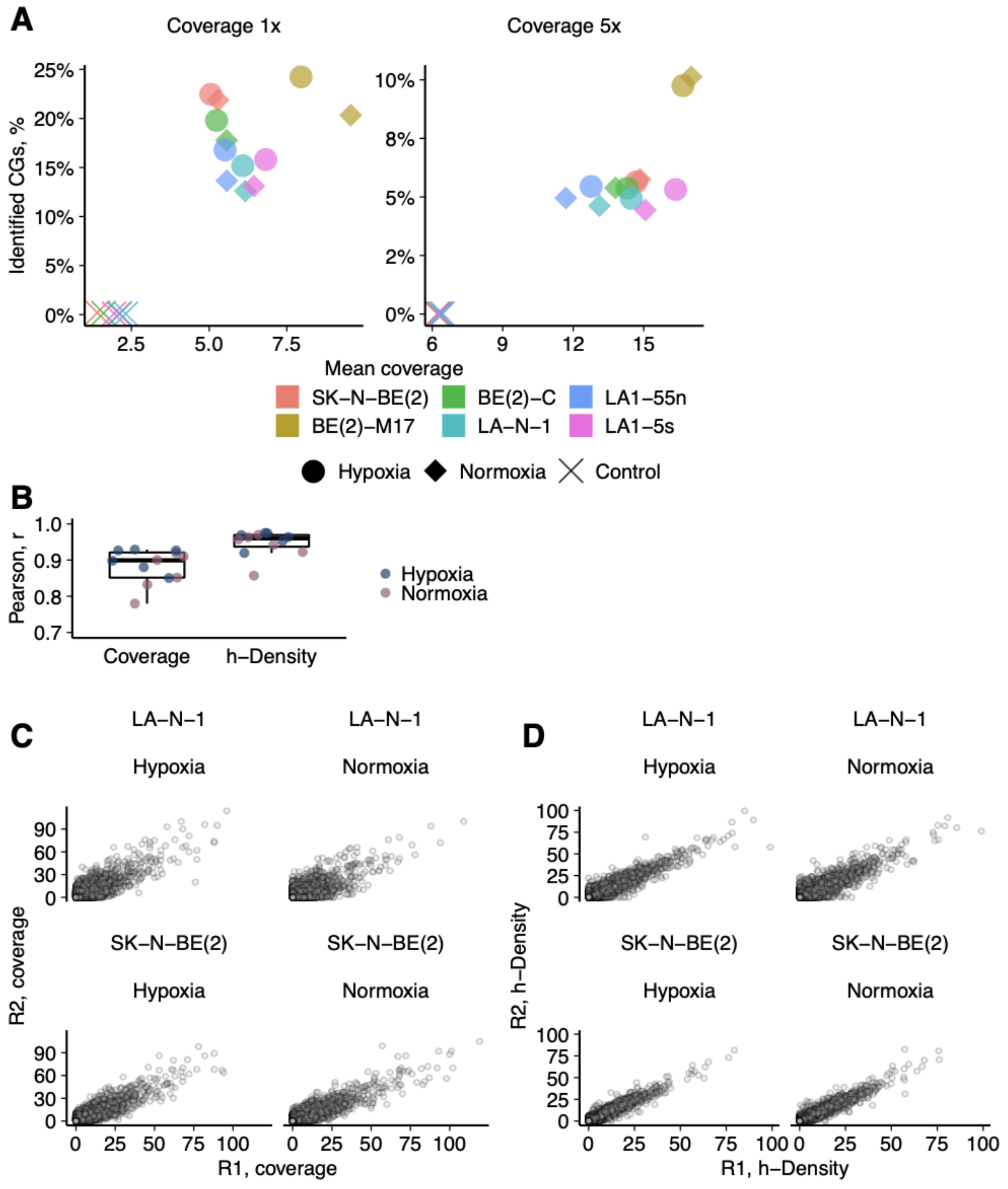

Supplementary Figure S3. Statistical parameters of hmTOP-seq analysis. **(A)** Mean coverage vs percentage of identified 5hmCG sites (of all ~28 M CG sites) in NB cell lines. For each cell type and condition a control library in which the BGT labeling step was omitted was prepared. The control libraries generated very low amounts of reads and did not correlate between themselves (Pearson mean r = 0.035, sd = 0.013), nor showed any correlation with the target 5hmCG signal (Pearson mean r = 0.041, sd = 0.014), indicating that the background signal corresponds to a random noise and cannot influence the target signal in a systematic way. Circles and diamonds represent hypoxic and normoxic NB cells,

respectively. **(B)** Correlation between technical replicates of the hmTOP-seq libraries of NB cells using hmTOP-seq coverage and h-density signals. **(C, D)** Scatter plots of 1e5 randomly selected 5hmCGs representing correlation between **(C)** mean coverage and **(D)** mean h-density of the hmTOP-seq library technical replicates for the combined signal of the SK-N-BE(2) and LA-N-1 group-related cells grown in atmospheric and hypoxic conditions.

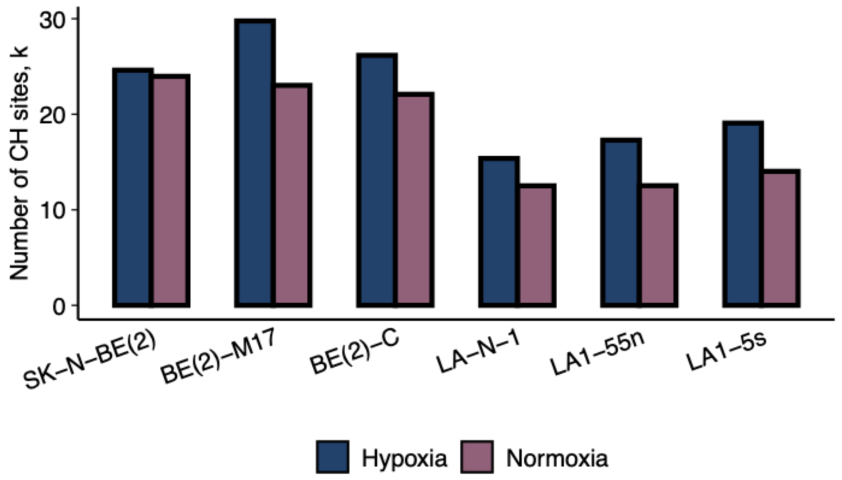

Supplementary Figure S4. Numbers (in thousands, k) of the identified hydroxymethylated CH sites in normoxic and hypoxic NB cells.

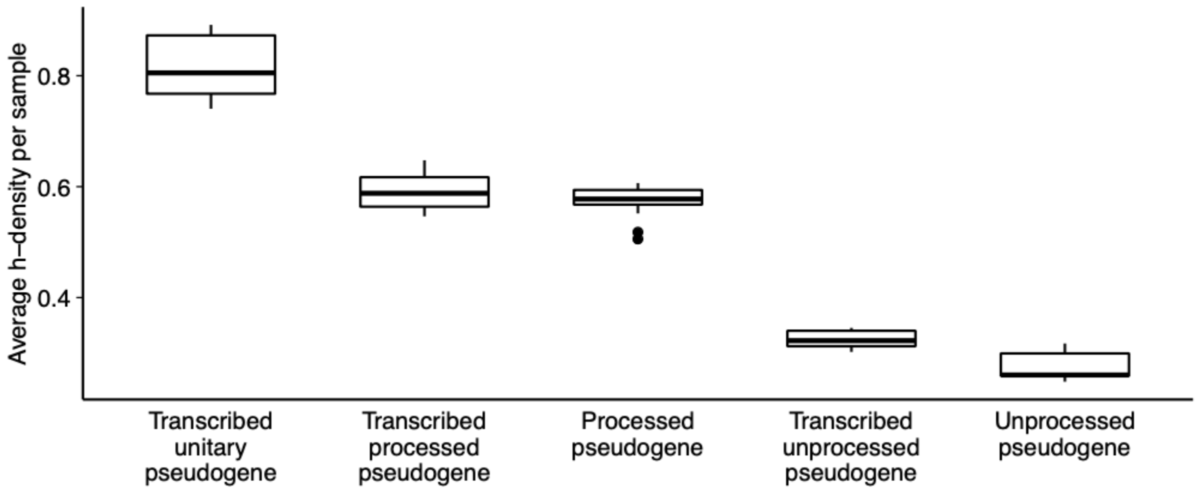

Supplementary Figure S5. Distribution of h-density across pseudogene groups (Pei et al. 2012) averaged for all NB cell lines.

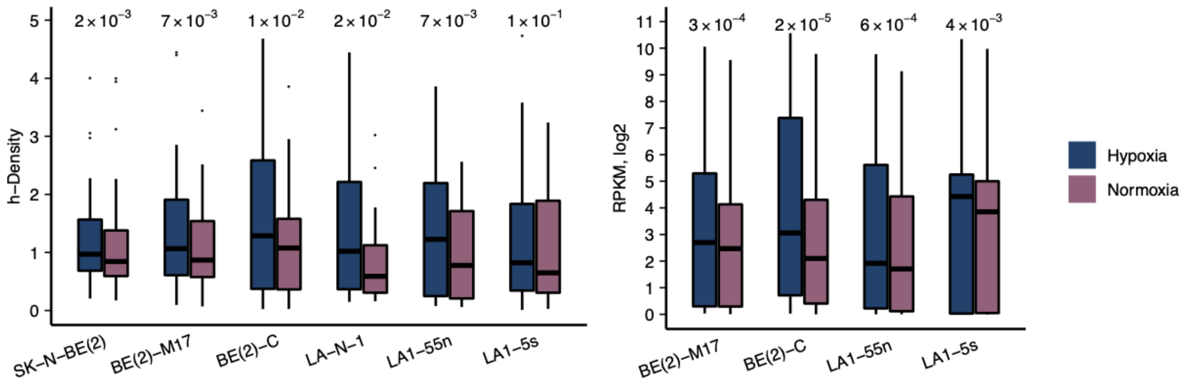

Supplementary Figure S6. Distribution of mean h-density and expression of 30 known hypoxia response genes in normoxic and hypoxic NB cells. P-values obtained using single-sided paired t-test.

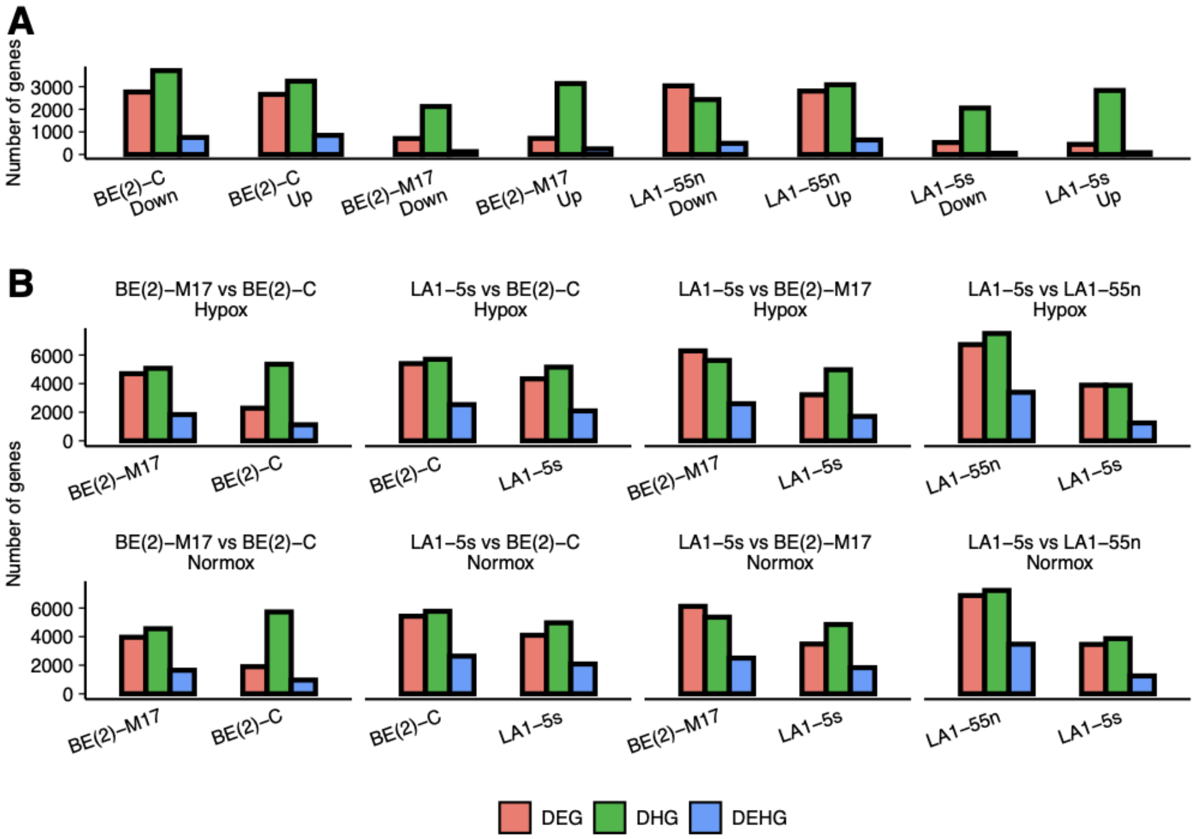

Supplementary Figure S7. Numbers of DHGs, DEGs and DEHGs identified in different NB cell lines. **(A)** Amounts of the hypoxia-affected Up and Down gene groups. **(B)** Amounts of the cell type-specific gene groups identified by pairwise comparison of hypoxic or normoxic NB cell lines.

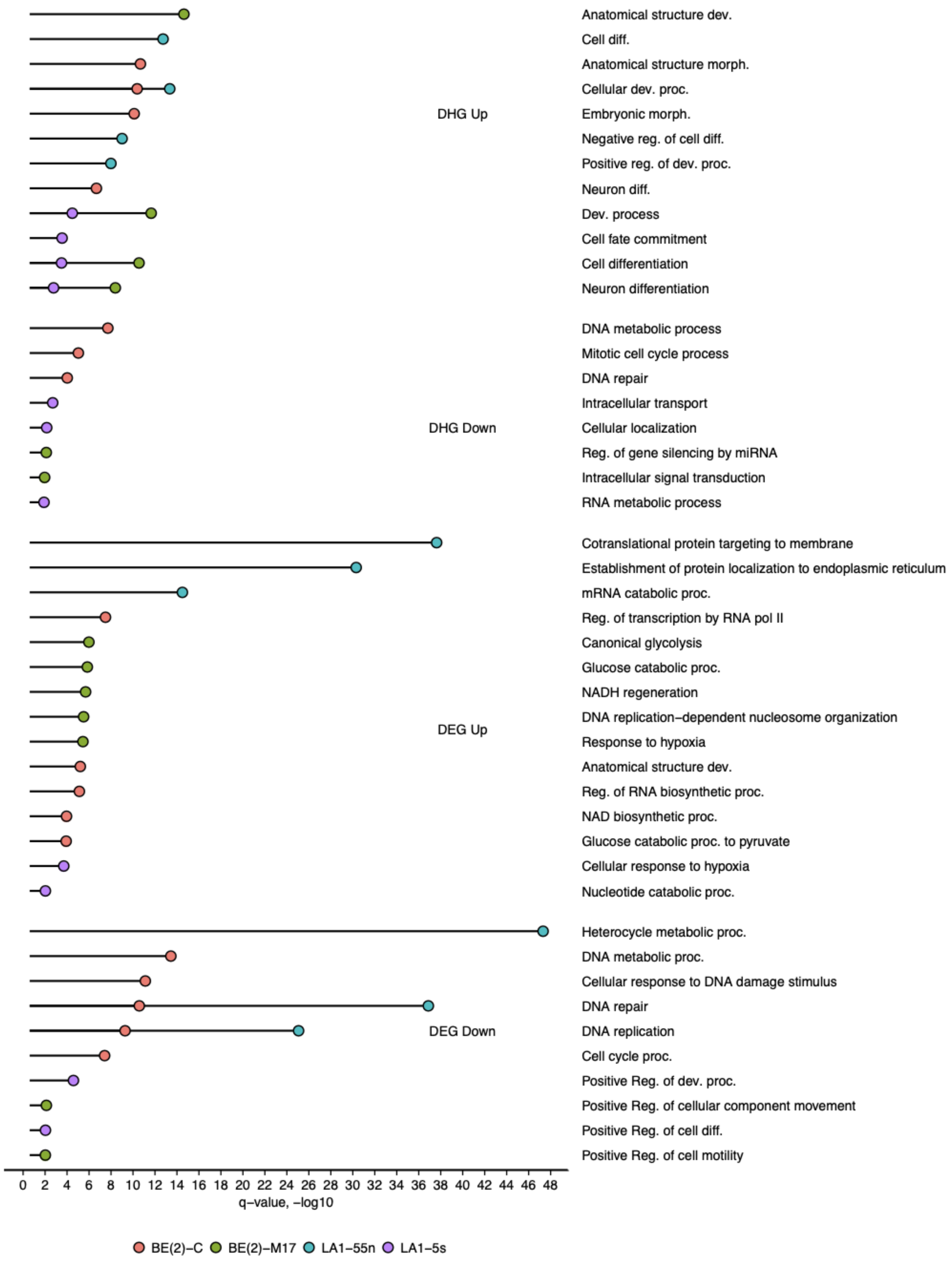

Supplementary Figure S8. GO functional annotation analysis of the hypoxia-affected Up and Down subgroups of DHGs and DEGs. Reg., regulation; Dev., development; Proc., process; Diff., differentiation; Morph., morphogenesis.

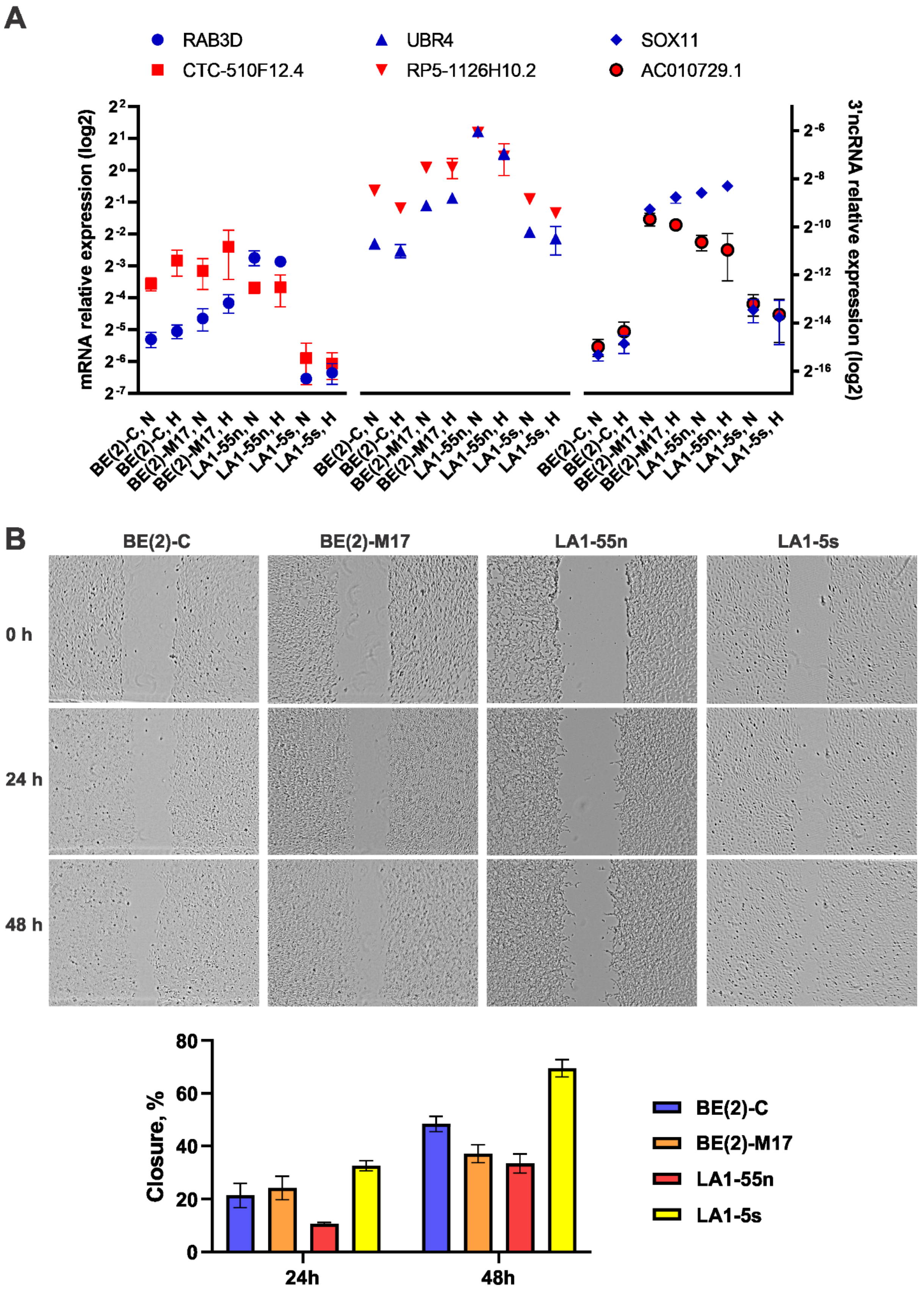

Supplementary Figure S9. RT-qPCR analysis of the most expressed (according to RNA-seq) 3'ncRNAs and their host genes and evaluation of the cell migration potential of NB cells. (A) Expression levels of 3'ncRNAs (*CTC-510F12.4*, *RP5-1126H10.2*

and *AC010729.1*) and their host gene (*RAB3D*, *UBR4* and *SOX11*, respectively) were determined by RT-qPCR after cultivating BE(2)-C, BE(2)-M17, LA1-55n and LA1-5s cells in atmospheric or hypoxic conditions for 72 h. The relative expression of each gene was calculated as $2^{-\triangle Ct}$ values and data represents mean ± sd (n=2). “N” and “H” define normoxia and hypoxia, respectively. **(B)** Wound healing assays were performed to determine the migration abilities of NB cells. Images were captured at 0, 24 and 48 h time points. Percentages of the wound closure are shown as mean ± sd from three independent replicates. Representative images of BE(2)-C, BE(2)-M17, LA1-55n and LA1-5s cells are shown.

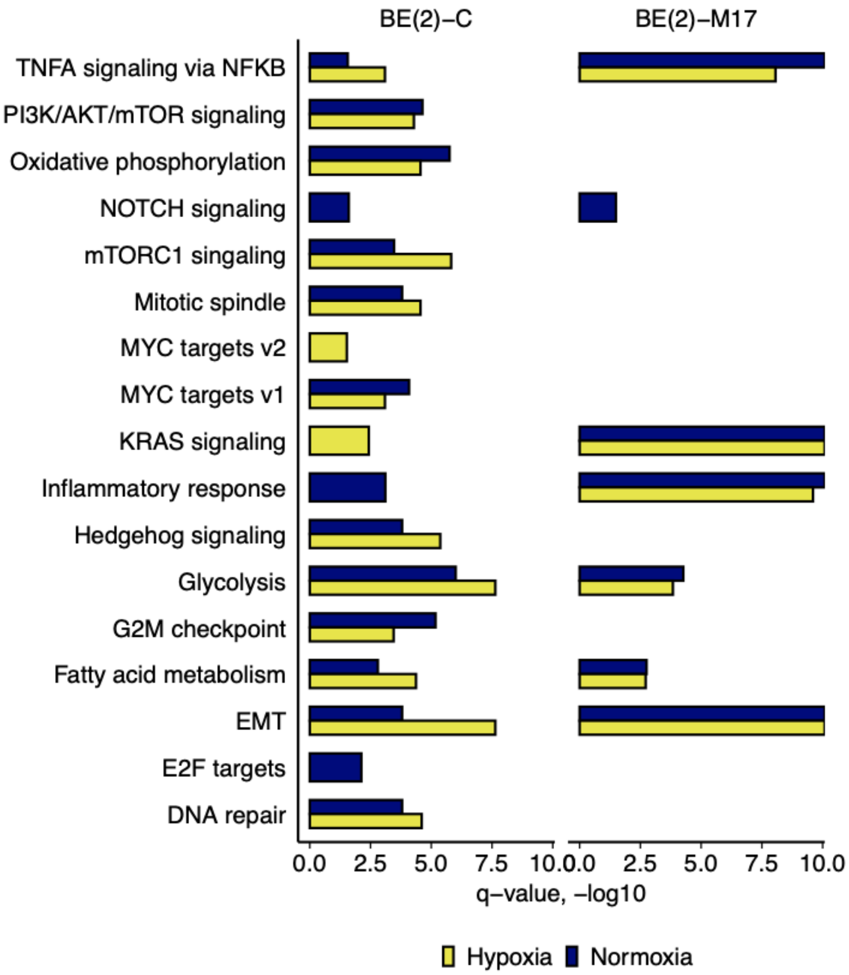

Supplementary Figure S10. Molecular pathway enrichment analysis using MSigDB Hallmark gene sets of the cell type-specific DHGs identified by comparing BE(2)-C (I-type) and BE(2)-M17 (N-type) cells, in normoxia and hypoxia. Significance of enrichment is represented with q-value.

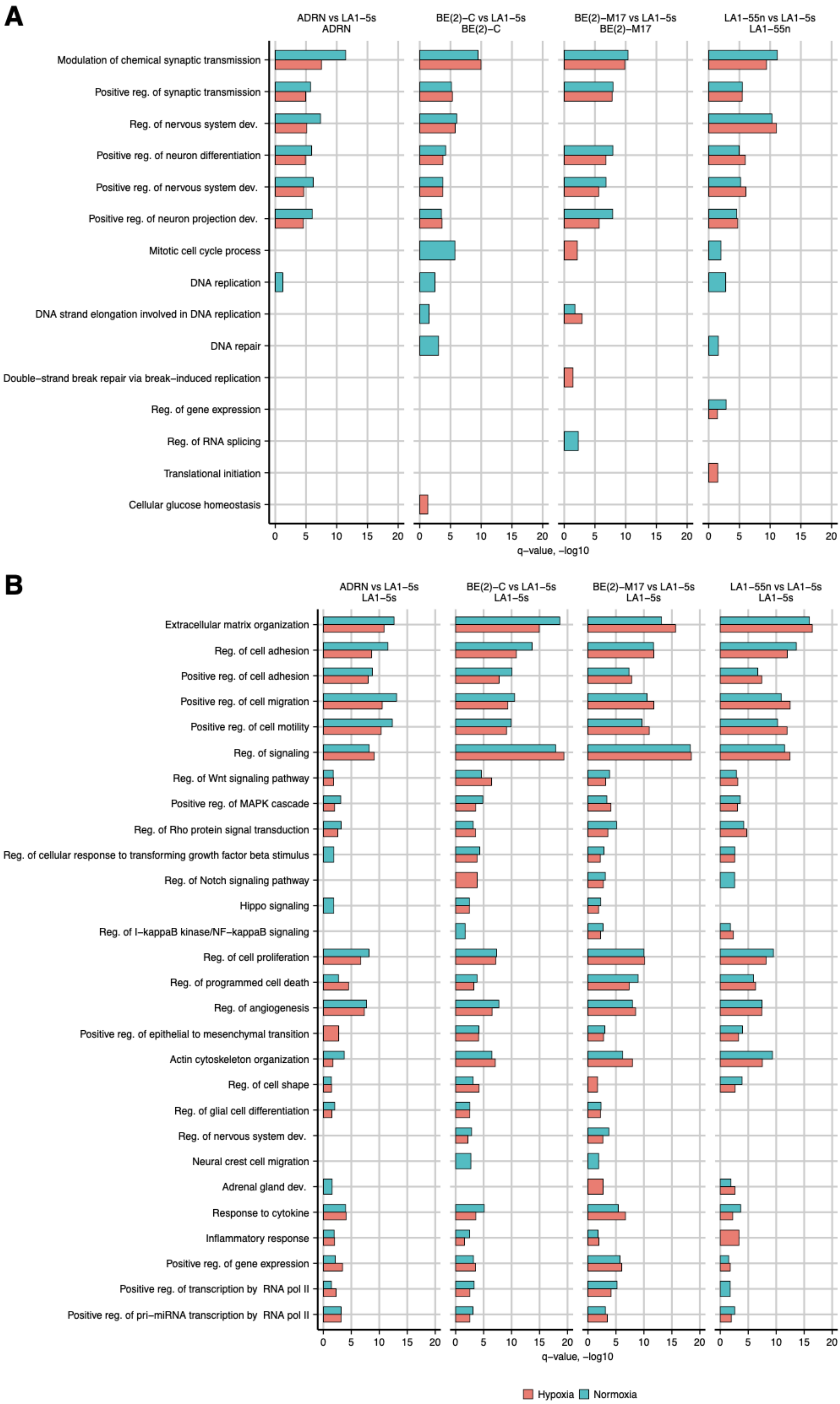
A
ADRN vs LA1−5s ADRN
BE(2)−C vs LA1−5s BE(2)−C
BE(2)−M17 vs LA1−5s BE(2)−M17
LA1−55n vs LA1−5s LA1−55n
Modulation of chemical synaptic transmission
Positive reg. of synaptic transmission
Reg. of nervous system dev.
Positive reg. of neuron differentiation
Positive reg. of nervous system dev.
Positive reg. of neuron projection dev.
Mitotic cell cycle process
DNA replication
DNA strand elongation involved in DNA replication
DNA repair
Double−strand break repair via break−induced replication
Reg. of gene expression
Reg. of RNA splicing
Translational initiation
Cellular glucose homeostasis
0 5 10 15 20
q−value, −log10
B
ADRN vs LA1−5s LA1−5s
BE(2)−C vs LA1−5s LA1−5s
BE(2)−M17 vs LA1−5s LA1−5s
LA1−55n vs LA1−5s LA1−5s
Extracellular matrix organization
Reg. of cell adhesion
Positive reg. of cell adhesion
Positive reg. of cell migration
Positive reg. of cell motility
Reg. of signaling
Reg. of Wnt signaling pathway
Positive reg. of MAPK cascade
Reg. of Rho protein signal transduction
Reg. of cellular response to transforming growth factor beta stimulus
Reg. of Notch signaling pathway
Hippo signaling
Reg. of I−kappaB kinase/NF−kappaB signaling
Reg. of cell proliferation
Reg. of programmed cell death
Reg. of angiogenesis
Positive reg. of epithelial to mesenchymal transition
Actin cytoskeleton organization
Reg. of cell shape
Reg. of glial cell differentiation
Reg. of nervous system dev.
Neural crest cell migration
Adrenal gland dev.
Response to cytokine
Inflammatory response
Positive reg. of gene expression
Positive reg. of transcription by RNA pol II
Positive reg. of pri−miRNA transcription by RNA pol II
0 5 10 15 20
q−value, −log10
Hypoxia
Normoxia

Supplementary Figure S11. GO functional annotation analysis is shown for DEHGs of **(A)** all ADRN cells combined, (BE(2)-C, BE(2)-M17 and LA1-55n), and **(B)** the MES/NCC-like LA1-5s cells grown in atmospheric and hypoxic conditions. The cell-type specific DEHGs were identified by comparing each of the cell lines ascribed to ADRN identity (BE(2)-C, BE(2)-M17 and LA1-55n) separately or combined, against the MES/NCC-like LA1-5s cells. Significance of enrichment is represented with q-value. Reg., regulation; Dev., development.

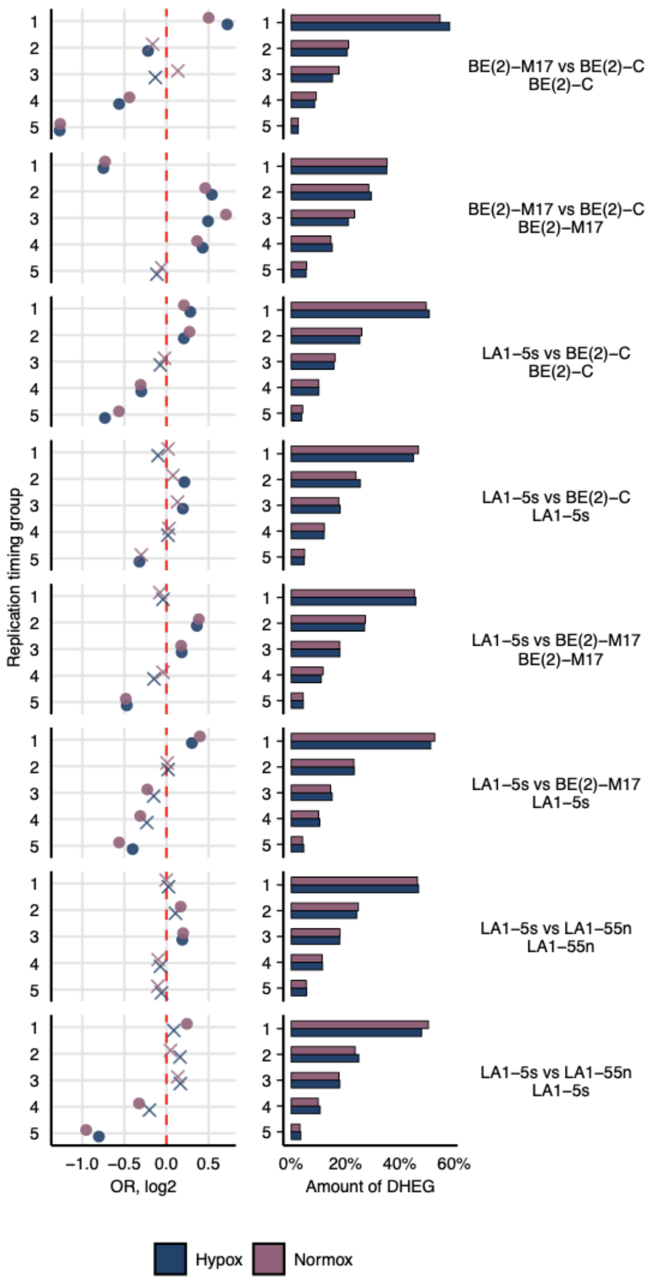

Supplementary Figure S12. Percentage and OR values from Fisher's exact test for enrichment of the cell type-specific DEHGs across the genomic regions which were assigned numbers from 1 to 5 based on replication timing (group 1 and 5 represent early and late replication timing groups, respectively). Non-significant estimates ($p \geq 0.05$) are marked with "X". Replication timing data of the NB cell line SK-N-SH was used for this graph (ENCODE Project; Thurman et al. 2007).

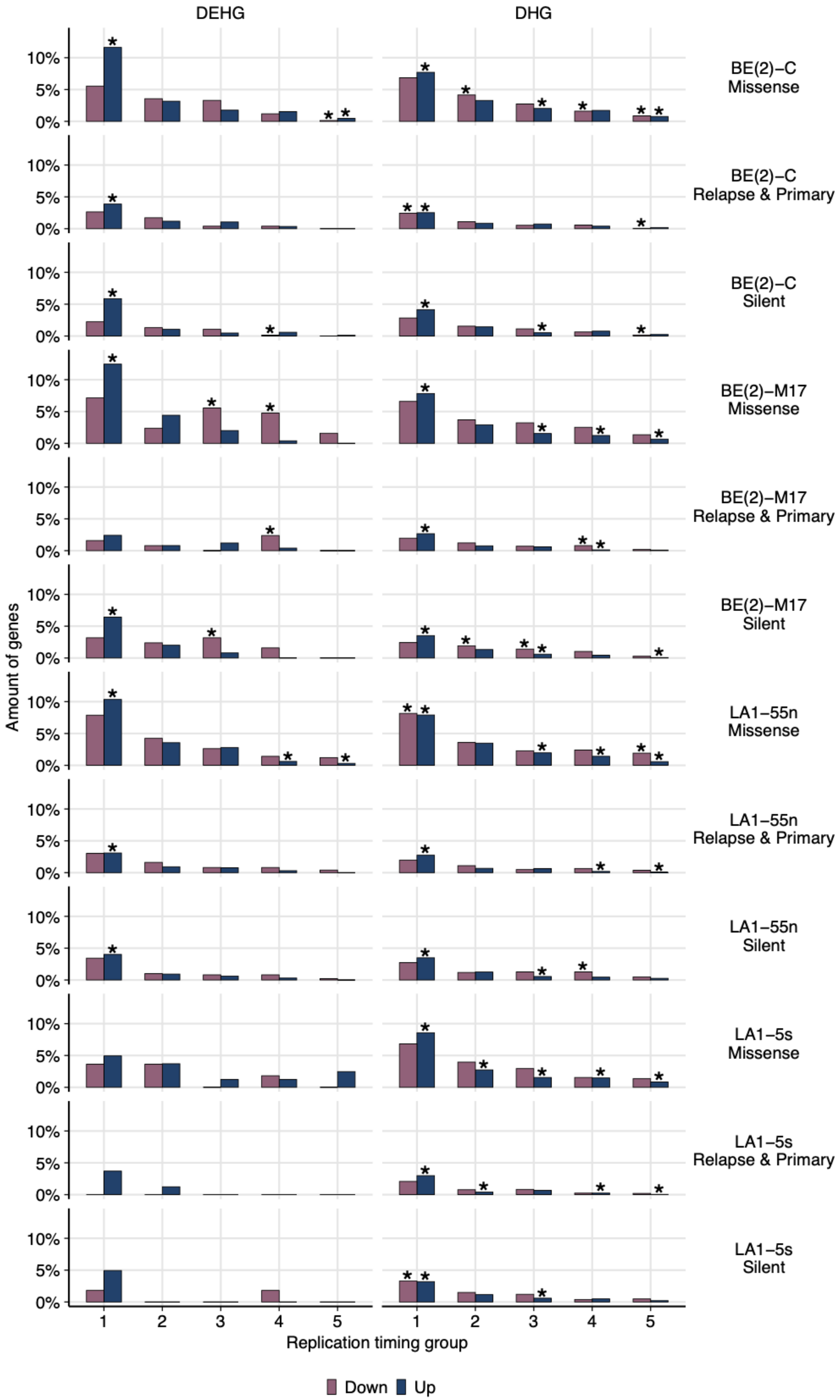

DEHG
DHG
BE(2)−C Missense
BE(2)−C Relapse & Primary
BE(2)−C Silent
BE(2)−M17 Missense
BE(2)−M17 Relapse & Primary
BE(2)−M17 Silent
LA1−55n Missense
LA1−55n Relapse & Primary
LA1−55n Silent
LA1−5s Missense
LA1−5s Relapse & Primary
LA1−5s Silent
Amount of genes
0%
5%
10%
1
2
3
4
5
Replication timing group
Down
Up

Supplementary Figure S13. Percentage of the hypoxic Up and Down DEHGs and DHGs overlapping SNVs from three different sets reported in NB tumours across genomic regions partitioned according to replication timing (group 1 and 5 represents early and late replicating regions, respectively). Asterix above the bars mark significant enrichments (Fisher's test p-value < 0.05). Replication timing data of the NB cell line SK-N-SH was used for this graph (ENCODE Project; Thurman et al. 2007).

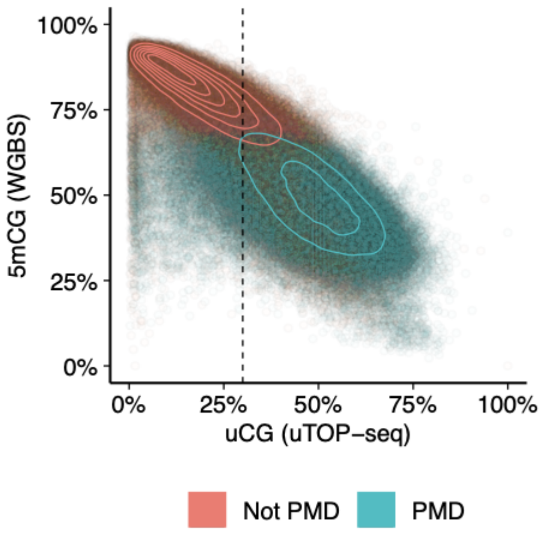

Supplementary Figure S14. Concordance between uTOP-seq uCG fractions and WGBS 5mCG methylation values in IMR90. The fractions of the identified uCG sites and CG methylation data (WGBS, Lister et al. 2009) were calculated across the 10-kb bins genome-wide. Vertical line at 30%-uCGs represents a threshold used to classify regions as PMDs.

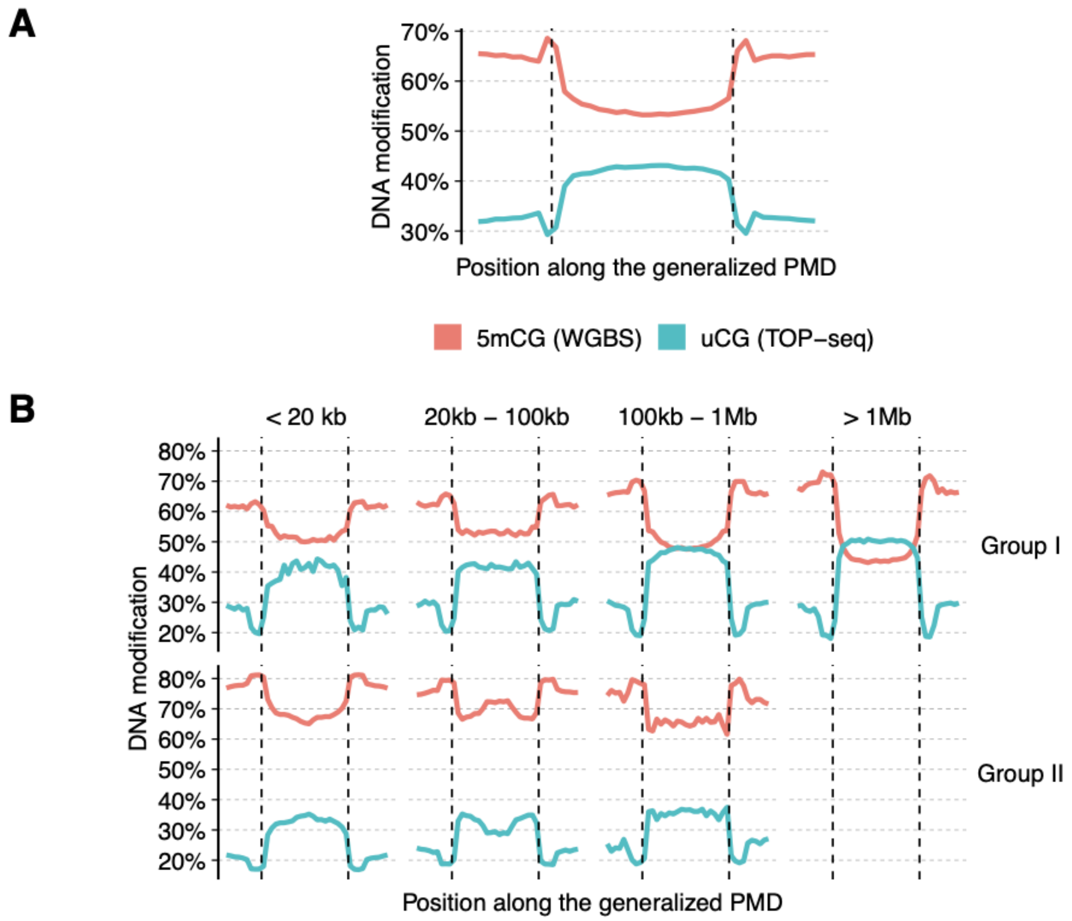

Supplementary Figure S15. Profiles of PMDs in IMR90 cells. (A) Profiles of uTOP-seq uCGs and WGBS 5mCGs across a generalized PMD region and 50-kb upstream and downstream regions. PMDs and upstream and downstream regions are divided into twenty equally sized non-overlapping windows. **(B)** uTOP-seq uCG and WGBS 5mCG profiles across PMDs divided into different size groups. PMDs showing lower and higher methylation levels are presented by Group I and Group II, respectively. uTOP-seq identified Group II PMDs with relatively higher CG methylation inside and outside of PMDs were not included in the reported PMD list of IMR90 (Lister et al. 2009).

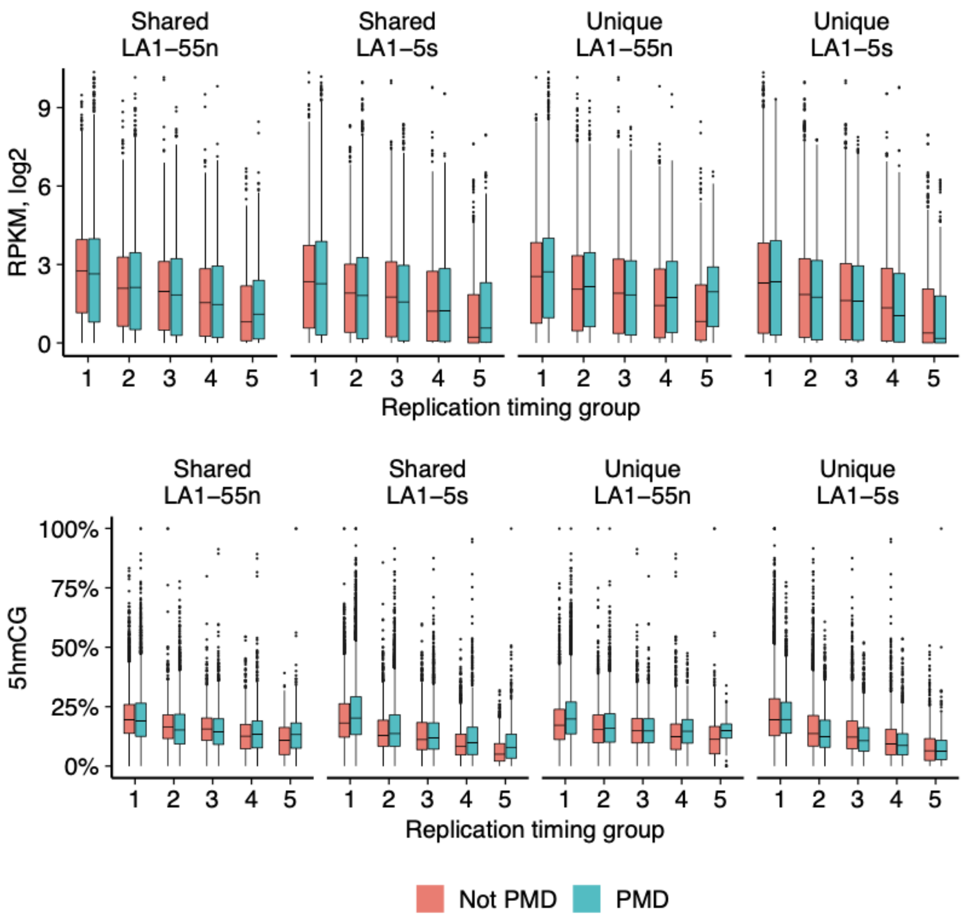

Supplementary Figure S16. Distribution of expression levels and 5hmCGs in all expressed genes in PMD and not PMD regions in relation to replication timing. Group 1 and 5 represent early and late replication timing groups, respectively. Replication timing data of the NB cell line SK-N-SH was used for this graph (ENCODE Project; Thurman et al. 2007).

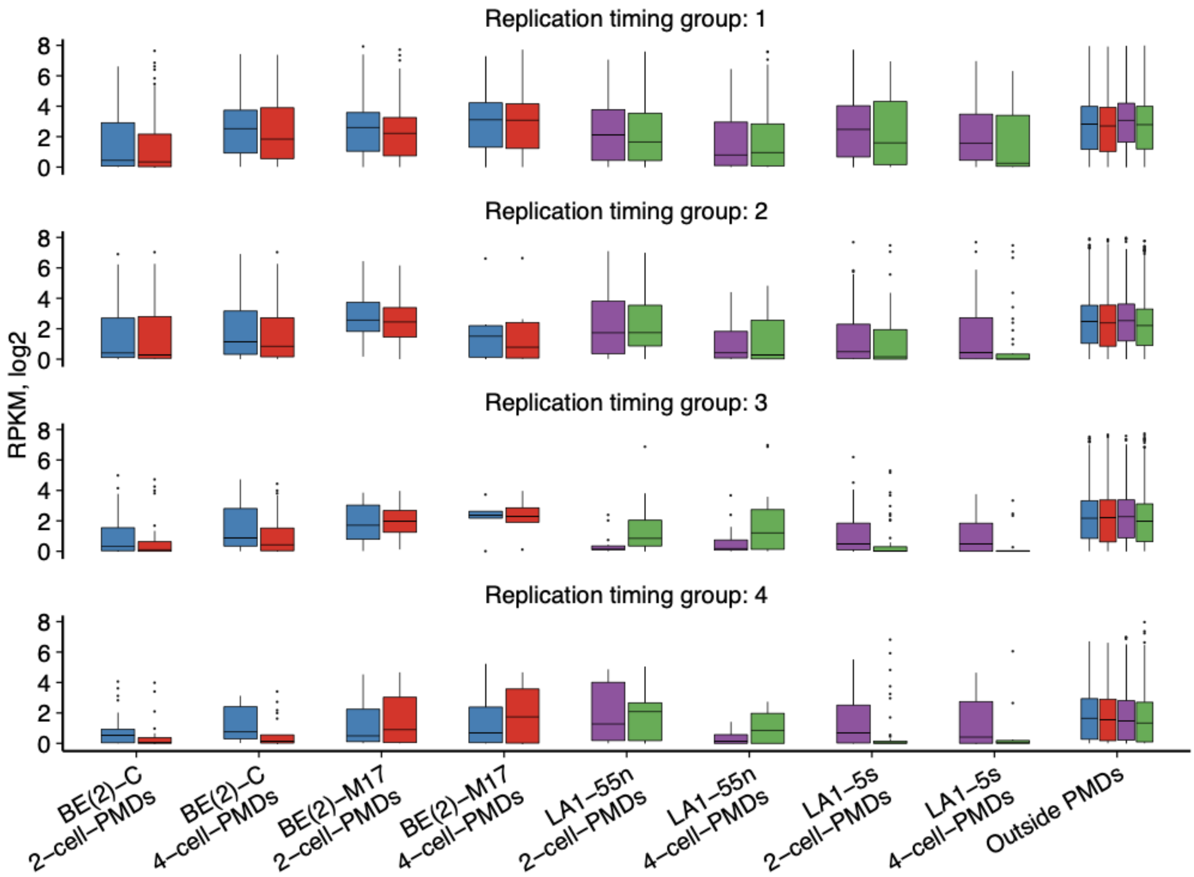

Supplementary Figure S17. Distribution of gene expression levels in the cell type specific unique 2-cell- and 4-cell-PMDs in relation to replication timing for the subpopulations of the SK-N-BE(2) and LA-N-1 cell groups, BE(2)-M17 and BE(2)-C, and LA1-55n and LA1-5s. Genomic regions were assigned numbers from 1 to 4 based on replication timing (1 and 4 represent early and late replication timing groups, respectively; in this graph, the fourth and fifth groups used throughout the study were joined due to the low number of genes in the group 5).

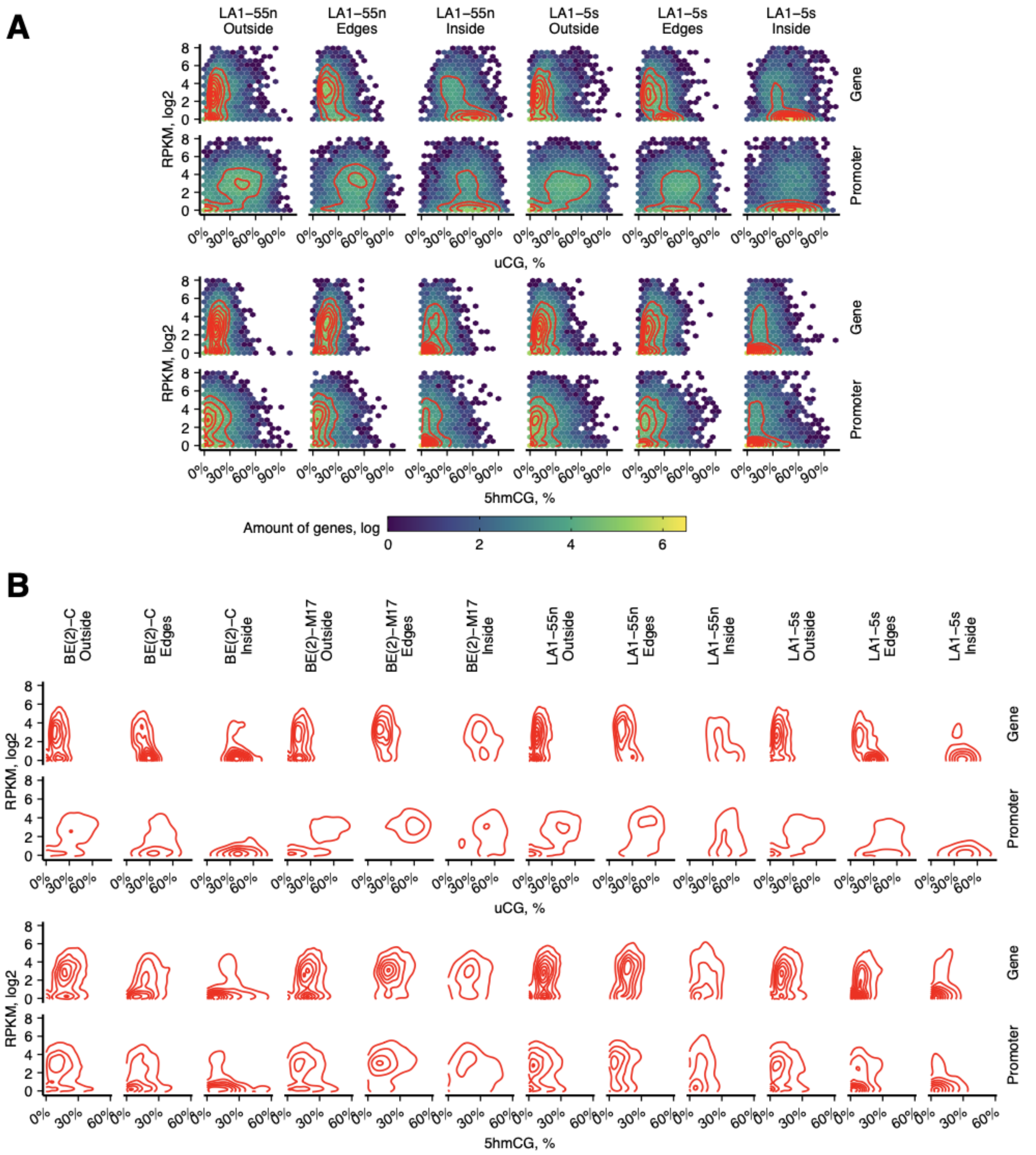

Supplementary Figure S18. (A) Heatmap plots and 2-dimensional density estimates for uCG/5hmCG fraction and expression data distribution for genes localized outside of PMDs (Outside), within PMDs (Inside), or crossing the boundaries of PMDs (Edges) in LA1-55n and LA1-5s. **(B)** 2-dimensional density estimates of uCG/5hmCG/RNA data for Outside, Inside and Edges-groups of the unique 2-cell-PMDs identified between BE(2)-M17 and BE(2)-C, or LA1-55n and LA1-5s.

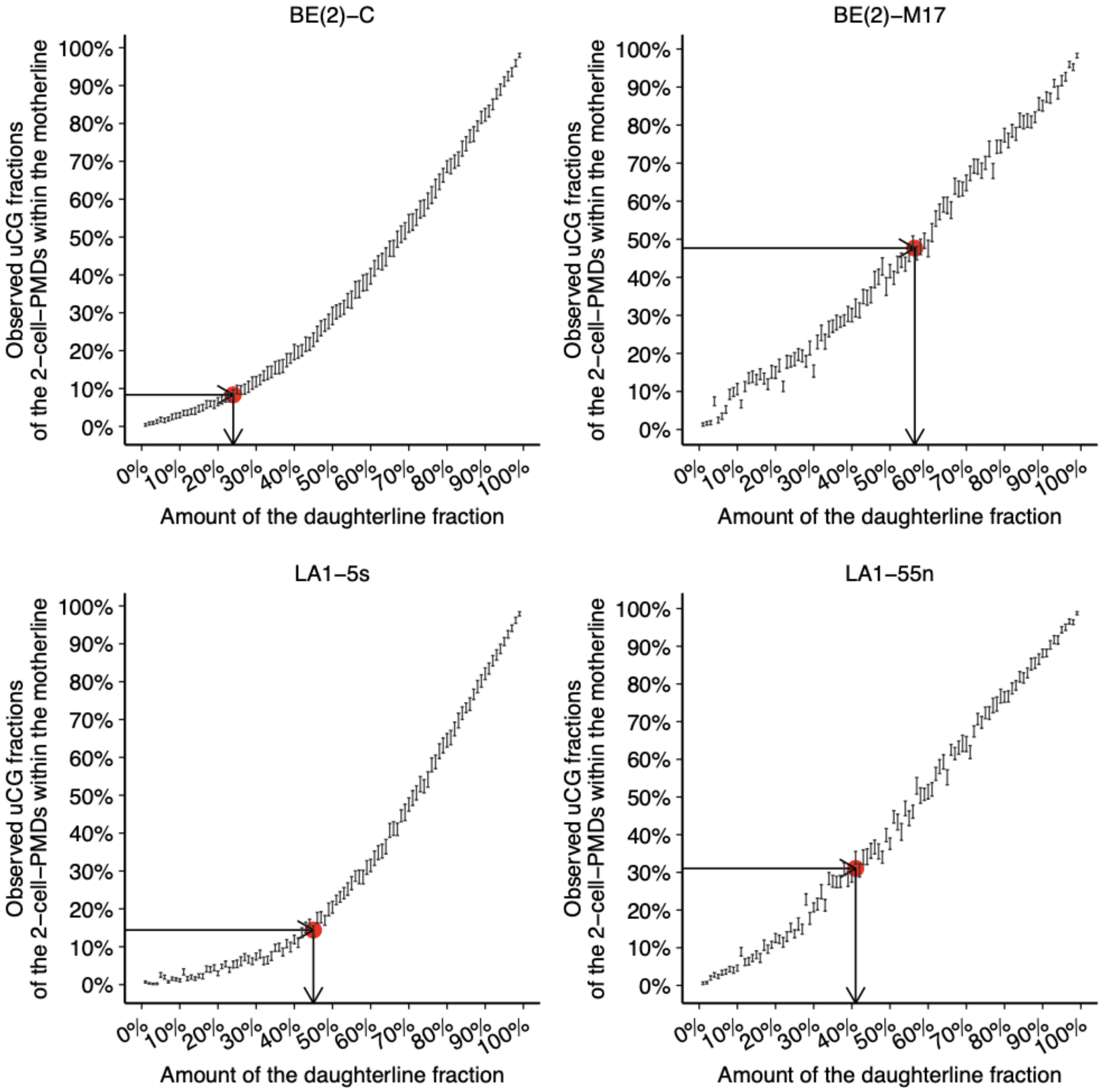

Supplementary Figure S19. Identification of the proportions of the subpopulations within the heterogeneous SK-N-BE(2) and LA-N-1 cell lines using cell type-specific PMDs. For construction of calibration curves, the cellular mixtures were *in silico* created by swapping CG identification status between BE(2)-M17 and BE(2)-C, or LA1-55n and LA1-5s at different ratios and *de novo* PMD regions were identified. Then, the fractions of uCG sites from the 2-cell-PMD regions were evaluated in each mixture. The *x*-axis specifies fractions of the daughterline subpopulation in the cellular mixtures, and the y-axis shows uCG-fractions of the unique 2-cell-PMDs. The calibration curves show a nearly linear relationship between the daughterline fractions in the mixtures and uCG-fractions in the unique 2-cell-PMDs. Red dot indicates the observed uCG-fraction of the unique 2-cell PMDs of the daughterline within the original motherline PMDs that predicts the fraction of the daughterline subpopulation within the motherline NB cell lines.

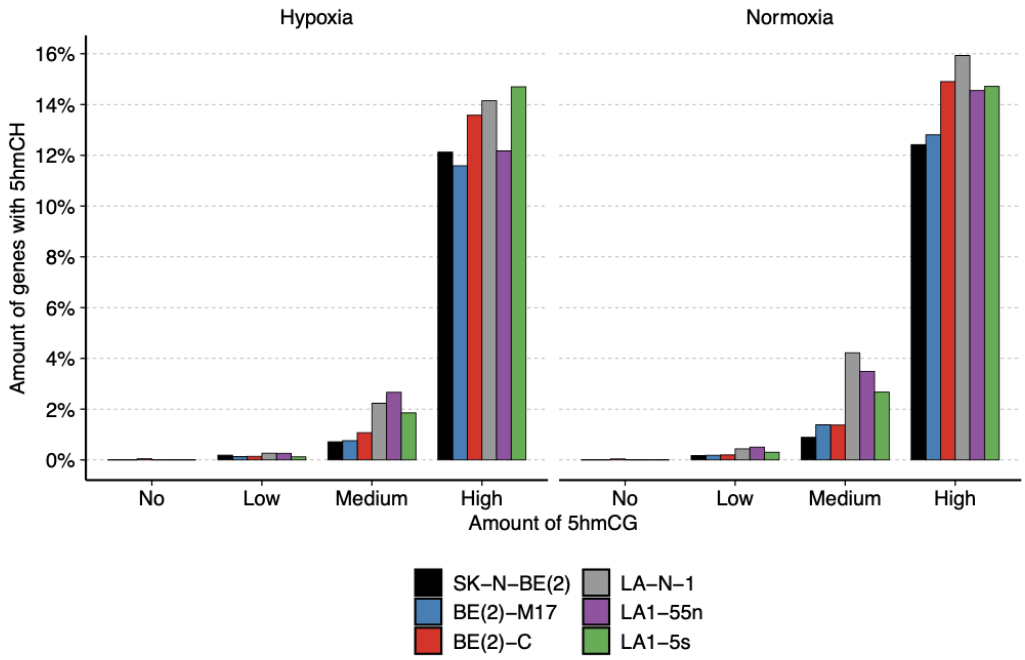

Supplementary Figure S20. Relation between gene 5hmCG and 5hmCH amounts. Barplots represent the amount of genes enriched in 5hmCHs as a function of the 5hmCG level in a gene. Genes containing 5hmCGs were grouped into three equally sized groups and the percentage of genes with 5hmCHs were computed for each NB cell line and condition.

Supplementary Tables

Supplementary Table S1. hmTOP-seq and uTOP-seq sequencing statistics.
Supplementary Table S2. Gene list of the hypoxia upregulated and downregulated genes in the SK-N-BE(2) and LA-N-1 groups.
Supplementary Table S3. The list of RT-qPCR primers.

References

Holmquist-Mengelbier, L., Fredlund, E., Löfstedt, T., Noguera, R., Navarro, S., Nilsson, H., et al. (2006). Recruitment of HIF-1alpha and HIF-2alpha to common target genes is differentially regulated in neuroblastoma: HIF-2alpha promotes an aggressive phenotype. Cancer Cell 10, 413–23. doi:10.1016/j.ccr.2006.08.026.

Jögi, A., Vallon-Christersson, J., Holmquist, L., Axelson, H., Borg, Å., and Påhlman, S. (2004). Human neuroblastoma cells exposed to hypoxia: induction of genes associated with growth, survival, and aggressive behavior. Exp. Cell Res. 295, 469–487. doi:10.1016/j.yexcr.2004.01.013.

Lister, R., Pelizzola, M., Dowen, R. H., Hawkins, R. D., Hon, G., Tonti-Filippini, J., et al. (2009). Human DNA methylomes at base resolution show widespread epigenomic differences. Nature 462, 315–322. doi:10.1038/nature08514.

Pei, B., Sisu, C., Frankish, A., Howald, C., Habegger, L., Mu, X., et al. (2012). The GENCODE pseudogene resource. Genome Biol. 13, R51. doi:10.1186/gb-2012-13-9-r51.

Thurman, R. E., Day, N., Noble, W. S., and Stamatoyannopoulos, J. A. (2007). Identification of higher-order functional domains in the human ENCODE regions. Genome Res. 17, 917–927. doi:10.1101/gr.6081407.
